# Structure-Based Virtual Screening of Ultra-Large Library Yields Potent Antagonists for a Lipid GPCR

**DOI:** 10.3390/biom10121634

**Published:** 2020-12-03

**Authors:** Arman A. Sadybekov, Rebecca L. Brouillette, Egor Marin, Anastasiia V. Sadybekov, Aleksandra Luginina, Anastasiia Gusach, Alexey Mishin, Élie Besserer-Offroy, Jean-Michel Longpré, Valentin Borshchevskiy, Vadim Cherezov, Philippe Sarret, Vsevolod Katritch

**Affiliations:** 1 Michelson Center for Convergent Biosciences, Department of Quantitative and Computational Biology, University of Southern California, Los Angeles, CA 90089, USA; big2ben@gmail.com; 2 Department of Chemistry, Bridge Institute, University of Southern California, Los Angeles, CA 90089, USA; asadybek@usc.edu (A.V.S.); cherezov@usc.edu (V.C.); 3 Department of Pharmacology-Physiology, Faculty of Medicine and Health Sciences, Institut de Pharmacologie de Sherbrooke, Université de Sherbrooke, Sherbrooke, QC J1H 5N4, Canada; Rebecca.Brouillette@USherbrooke.ca (R.L.B.); Elie.Besserer-Offroy@USherbrooke.ca (É.B.-O.); Jean-Michel.Longpre@USherbrooke.ca (J.-M.L.); Philippe.Sarret@USherbrooke.ca (P.S.); 4 Research Center for Molecular Mechanisms of Aging and Age-Related Diseases, Moscow Institute of Physics and Technology, 141701 Dolgoprudny, Russia; marin@phystech.edu (E.M.); snurka88@gmail.com (A.L.); anastasia.gusach@gmail.com (A.G.); mishinalexej@gmail.com (A.M.); borshchevskiy@gmail.com (V.B.); 5 Department of Pharmacology and Therapeutics, McGill University, Montreal, QC H3G 1Y6, Canada; 6 Institute of Biological Information Processing (IBI-7: Structural Biochemistry), Forschungszentrum Jülich GmbH, 52425 Jülich, Germany; 7 JuStruct: Jülich Center for Structural Biology, Forschungszentrum Jülich GmbH, 52425 Jülich, Germany

**Keywords:** structure-based lead discovery, virtual ligand screening, GPCR, cysteinyl leukotriene, CysLT receptors, asthma, uveal melanoma

## Abstract

Cysteinyl leukotriene G protein-coupled receptors, CysLT1R and CysLT2R, regulate bronchoconstrictive and pro-inflammatory effects and play a key role in allergic disorders, cardiovascular diseases, and cancer. CysLT1R antagonists have been widely used to treat asthma disorders, while CysLT2R is a potential target against uveal melanoma. However, very few selective antagonist chemotypes for CysLT receptors are available, and the design of such ligands has proved to be challenging. To overcome this obstacle, we took advantage of recently solved crystal structures of CysLT receptors and an ultra-large Enamine REAL library, representing a chemical space of 680 M readily available compounds. Virtual ligand screening employed 4D docking models comprising crystal structures of CysLT1R and CysLT2R and their corresponding ligand-optimized models. Functional assessment of the candidate hits yielded discovery of five novel antagonist chemotypes with sub-micromolar potencies and the best Ki = 220 nM at CysLT1R. One of the hits showed inverse agonism at the L129Q constitutively active mutant of CysLT2R, with potential utility against uveal melanoma.

## 1. Introduction

Cysteinyl leukotrienes (CysLTs) are lipid-like inflammatory mediators produced via the 5-lipoxygenase (5-LO) pathway. They activate two subtypes of CysLT G-protein-coupled receptors (GPCRs), CysLT1R and CysLT2R, that share 38% sequence identity and signal mainly through the Gq/11 pathway [1], with additional coupling to Gi/o [2]. Both CysLT receptors respond to endogenous cysteinyl leukotrienes LTC4, LTD4, and LTE4; however, CysLT1R has some preference for LTD4, while CysLT2R has a higher affinity towards LTC4 [1]. Since CysLTRs are involved in inflammatory processes, they play an important role in the development of asthma, allergic rhinitis, and cardiovascular diseases [1,3,4,5]. CysLT1R is mainly expressed in smooth muscle cells and macrophages, while CysLT2R has a broader expression profile in immune cells [6]. Recently, a single nucleotide polymorphism L129Q in human CysLT2R was discovered as a driver oncogenic mutation leading to uveal melanoma [7] and potentially some other melanocytic tumors [8,9,10]. Further studies suggested that this mutation causes constitutive CysLT2R activation with a high bias towards Gq signaling, which was not effectively inhibited by known CysLT antagonists [11].

Despite their clinical importance, few drugs are known for CysLTRs. Selective CysLT1R antagonists, such as montelukast, zafirlukast, and pranlukast, are often used for the treatment of asthma and allergic rhinitis. However, in a large fraction of patients, the efficacy of these medications is limited [12]. Additionally, gastrointestinal symptoms and neuropsychiatric side effects have been reported for these ligands [13,14]. No antagonists for CysLT2R are used in clinic or are currently under study in clinical trials, although animal asthma models suggested that dual CysLT1R/CysLT2R antagonists may be considered for treatment of severe cases of asthma [15]. In addition, recent preclinical studies showed that a CysLTR antagonist, quininib, with a micromolar IC_50_ against CysLTRs, represents a promising regulator of angiogenesis in cancer, which outlines the importance of dual CysLT1R/CysLT2R antagonist activities [16,17].

In the past decade, the increasing numbers of high-resolution GPCR structures, along with advances in computer-assisted drug discovery, have led to numerous successful virtual ligand screening (VLS) campaigns for GPCRs, including the discovery of novel nanomolar ligands for dopamine D4 and melatonin MT1 and MT2 receptors [18,19,20,21]. Yet, no successful structure-based virtual ligand screenings have been reported for a lipid GPCR, even though structures of more than a dozen of lipid receptors became available since 2012 [22,23], suggesting that this type of receptor is especially challenging for VLS.

Most recently, the utility of virtual ligand screening for lead discovery has also been boosted by the expansion of accessible chemical space through make-on-demand compound libraries like Enamine REAL library [24]. These virtual libraries currently cover more than 190 parallel one-pot reaction procedures that use over 113,000 qualified reagents and enumerate about 680 Million make-on-demand compounds. Utilization of such libraries in virtual ligand screenings [19,20] or their diversity subsets of 100–200 million compounds demonstrated high success rate in hit determination and can streamline further hit-to-lead optimization.

Last year, we published high-resolution crystal structures of CysLT1R with zafirlukast and pranlukast [25], and CysLT2R with several non-selective antagonists [26]. Analysis of the structures and accompanying biochemical studies have provided insights into ligand selectivity, unusual activation mechanism, and distinct sodium binding features in CysLT1R. All complexes, except CysLT1R-zafirlukast, have shown similar binding poses with negatively charged groups bound within the seven transmembrane (7TM) bundle and lipophilic tail located between TM4 and TM5. For zafirlukast, we observed an induced-fit binding mode that involves TM5 extracellular tip movement, allowing ligand entry into the pocket directly from the lipidic membrane. Notably, the CysLT1R construct used for structure determination has no thermostabilizing mutations, while the three mutations used for CysLT2R are far from the ligand binding pocket, supporting the suitability of structures for docking and VLS.

Here, we report a large-scale structure-based virtual screening for CysLTRs ligands using a diversity subset of 115 million molecules from Enamine REAL lead-like and diverse drug-like libraries, representing chemical space of 680 million compounds. The multi-template 4D structural model [27] used for screening was built using CysLTR1 and CysLTR2 crystal structures, as well as ligand-guided optimized models. Testing of 139 selected hits revealed 10 and 15 potential lead compounds for CysLT1R and CysLT2R, respectively, with inhibition below 50% at 30 µM concentrations. The most interesting five compounds were further confirmed in functional assays for their CysLT1R antagonist activity at inhibiting LTD4 or LTC4-induced responses, with potencies in the submicromolar range. Most compounds showed CysLT1R selectivity, though BRI-12359 binds both receptors. Moreover, BRI-12359 and BRI-12417 demonstrated potent inverse agonism behavior against the constitutively active CysLT2R L129Q mutant involved in uveal melanoma.

## 2. Materials and Methods

### 2.1. Receptor Model Preparation and Optimization

X-ray crystal structures of CysLT1R and CysLT2R were employed in virtual ligand screening and preparation of ligand-optimized structural models. CysLT1R virtual screening models were prepared using the X-ray structure of CysLT1R with pranlukast at 2.7 Å resolution [25] (PDB ID 6RZ4). CysLT2R models were prepared using the X-ray structure of CysLT2R receptor with an antagonist ONO-2570366 at 2.4 Å resolution [26] (PDB ID 6RZ6). The structures were converted from PDB coordinates to ICM objects using an ICM-Pro conversion algorithm (www.molsoft.com). The conversion procedure involves building of hydrogen and missing heavy atoms, local minimization of polar hydrogens, optimization of His, Asn and Gln side-chain rotamers, assigning the protonation state and secondary structure.

To account for binding pocket flexibility upon binding of different ligand scaffolds, ligand-guided receptor optimization algorithm (LiBERO [28]) was used to refine the sidechains and water molecules in 8 Å radius from the orthosteric ligand. For validation of each of the models, two diverse sets (22 molecules each (Supplementary Tables S2 and S3) of known high-affinity antagonists (ChEMBL [29], pAct > 8) were docked into the corresponding models, and compared with 200 decoy compounds. The decoy compounds were selected as 50 diverse molecules from ChEMBL24.1, which are not active at CysLTRs (pAct < 5.5) and 150 diverse compounds from CysLT1R entry in GPCR Decoy Database [30] (Supplementary Table S4). The same set of decoys was used for CysLT2R. The ROC (Receiver Operating Characteristics) curves were used to quantitatively evaluate the models, where ROC curves were plotted based on the True Positive Rates (TPR) and False Positive Rates (FPR) from the docking of true binders and decoys. Both Area Under the Curve (AUC) and Normalized Squared AUC (NSA) values were used to select the best models [28]. The importance of the co-crystallized water molecules in the binding pocket was assessed based on their impact on the ROC curve. Only waters that improved the performance of the models were included in the final ligand-based optimized CysLT1R and CysLT2R models.

### 2.2. Screening Libraries

Two libraries from Enamine Real Database were used in virtual ligand screening: Diverse REAL drug-like and REAL 350/3 lead-like libraries. A diverse subset of REAL drug-like library of 15 million compounds represents the REAL drug-like space of 1.2 billion compounds and contains compounds that comply with the “rule of five”. REAL 350/3 lead-like library contains 100 million compounds that comply with the following rules: 270 ≤ MW ≤ 350, 14 ≤ heavy atoms ≤ 26, SlogP ≤ 3, and aryl rings ≤ 2. PAINS and toxic compounds were removed. All compounds were converted from SMILES to 3D format and formal charges were assigned at pH7 according to ICM pKa Model implemented in ICM-Pro [31].

### 2.3. Virtual Ligand Screening

Docking and VLS simulations were performed using ICM-Pro molecular modeling software (Molsoft LLC, version 3.8-7a). CysLT1R and CysLT2R crystal structures as well as their refined models were employed in 4D docking as described previously [27]. 4D docking allowed to screen the chemical library against two receptors (two models for each receptor) in a single docking run saving time and computational resources. The energy potential maps were calculated for each model and stored in a single multi-dimensional map file (4D grid). During docking, ligands were given full torsion flexibility in internal coordinates. Docking simulations used biased probability Monte Carlo (BPMC) optimization of the compound’s internal coordinates in the pre-calculated 4D grid energy potentials. Electrostatic energy, hydrophobic energy, internal ligand energy, protein-ligand hydrogen bonding energy, and desolvation energy terms were considered during the energy optimization and scoring of docked molecules, as described previously [32] and successfully implemented in several campaigns for GPCR targets with the high hit rate [18,21,33].

In VLS, the exhaustive sampling of the molecule conformational space in the rectangular box of the orthosteric binding pocket was performed and the best docking conformation of each molecule was stored with the corresponding predicted binding score.

After the 4D docking step, top 20,000 compounds were redocked twice with thoroughness increased to two into individual receptor models to improve predicting accuracy. Top compounds from each hitlist were merged to form a list of 2000 chemicals. The resulting compounds were filtered based on important interaction patterns in the binding pocket such as formation of hydrogen bonds with crucial residues. To ensure chemical diversity, the compounds were clustered based on Tanimoto distance and best scoring compounds were selected from each cluster. The chemical novelty of top hits was verified using the Tanimoto distance to known CysLT1R and CysLT2R ligands from ChEMBL database. Tanimoto distances were calculated as implemented in ICM-Pro [31], using linear and non-linear (ECFP) fragment enumeration to construct fingerprints.

### 2.4. IP1 Production Assay

All 139 selected compounds were screened in a functional assay measuring IP1 production as an indicator of Gαq activation. For this assay, we used the Cisbio IP-One kit as previously described [34]. Human Embryonic Kidney (HEK) 293 cells (ATCC CRL-1573) between passages 5 and 25 were seeded onto poly-L-Lysine-coated 384-well plates at 20,000 cells/well and transfected with 30 ng of cDNA coding for the wild-type CysLT1R or CysLT2R receptors or for the CysLT2R mutant L129Q, using the Lipofectamine 3000 (Thermo Fisher Scientific) transfection agent. At 48 h post-transfection, the media was removed and the cells were washed with fresh Hank’s Balanced Salt Solution (HBSS). Cells were then stimulated with compounds according to agonist (direct compound stimulation) or to antagonist (co-stimulation of compounds with an EC_80_ concentration of LTD4) experimental protocols. First, all 139 selected compounds were screened at a 30 µM concentration in both the agonist and antagonist mode in cells transiently expressing the wildtype CysLT1R or CysLT2R receptors. Potential antagonist hits (≥50% inhibition) were retained for further study. For CysLT1R, the compounds retained were BRI-12328, BRI-12334, BRI-12359, BRI-12410, BRI-12411, BRI-12417, BRI-12424, BRI-12435, BRI-12450, and BRI-12458; for CysLT2R, these were BRI-12328, BRI-12334, BRI-12359, BRI-12401, BRI-12402, BRI-12405, BRI-12410, BRI-413, BRI-12417, BRI-12424, BRI-426, BRI-427, BRI-450, BRI-453, BRI-454, BRI-458, and BRI-466. These hits were again tested in the antagonist mode with compound concentrations ranging from 1.56 µM to 100 µM, at 2-fold concentration intervals. The three best hits (BRI-12359, BRI-417, and BRI-424) were also tested using the LTC4 ligand (EC_80_ concentration) and in cells transiently expressing the constitutively active CysLT2R mutant L129Q. Finally, additional experiments were performed in which CysLT1R- or CysLT2R-expressing cells were pre-stimulated with fixed concentrations of BRI-12359 (1, 10, 30, 60 or 100 µM) and then stimulated with a range of LTD4 concentrations (10^−12^ M to 10^−6^ M). After compound stimulation, the cells were incubated for 1 h at 37 °C and stimulated with IP1-D2 and Ab-Crypt reagents in Lysis Buffer. The cells were then incubated at room temperature for an additional hour. The plate was read on a Tecan Genios Pro plate reader using a HTRF (homogeneous time-resolved fluorescence) filter set (λex = 320 nm, λem = 620 and 655 nm). Normalized data were plotted using either the three-parameter or four-parameter EC_50_/IC_50_ fits of GraphPad Prism 8 (San Diego). In those cases where reliable IC_50_ values could be obtained from the fits, we estimated functional inhibitory constants (K_i_) using the Cheng-Prusoff equation: K_i_ = IC_50_ × ([A]/EC_50_ + 1)^−1^, in which [A] is the fixed concentration used of the LTD4 or LTC4 agonist, and EC_50_ the concentration of this agonist that produces a half-maximal response at the receptor [35]. Data represent the means ± SEM of at least three independent experiments performed in quadruplicate. For the additional antagonist experiments performed with BRI-12359, we performed Schild analysis in order to determine pA2 values.

## 3. Results

### 3.1. Optimization of Receptor Models

Crystal structures of CysLT1R (PDB ID: 6ZR4, co-crystallized ligand pranlukast [25]) and CysLT2R (PDB ID: 6RZ6, co-crystallized ligand ONO-2570366 [26]) were used to prepare models for prospective virtual screening. We employed the ligand-guided receptor optimization algorithm, LiBERO [28], modified to include crystallographic water molecules, to refine conformations of sidechain in the binding pocket, within 8 Å of the co-crystallized ligands (see Methods). The best receptor models were selected based on the area under the receiver operator characteristic curve (AUC) and Normalize Squared AUC (NSA) values, which quantitate the model ability to correctly discriminate between ligands and decoys (Table 1, Supplementary Figure S1). In the final LiBERO-optimized models, we included only those water molecules that led to a better predictive model. Benchmarking showed that in the CysLT1R crystal structure, removal of all water molecules from the binding pocket led to the best docking results for active ligands. In cases with CysLT2R crystal structure and LiBERO-optimized CysLT1R and CysLT2R structures, benchmarking revealed that only one water molecule in each of these cases is important for the best discrimination between active and decoy molecules in docking. Thus, in the CysLT1R LiBERO-optimized structure, a water molecule in between residues Y26^1.35^, Y30^1.39^, R79^2.60^ and Y83^2.64^ was kept. In the CysLT2R crystal structure and LiBERO-optimized structure, water molecule in between residues T90^2.56^, R94^2.60^ and S117^3.31^ was present during VLS. The best LiBERO-optimized models showed reproducible and consistent poses of co-crystallized ligands, as well as dramatically improved ROC values and better predicted binding scores for the high-affinity ligands of CysLT1R and CyslT2R receptors (Table 1). The benchmarking results validated the predictive ability of the optimized models. CysLT1R and CysLT2R crystal structures and corresponding LiBERO-optimized models were used to generate a multi-template structural model for 4D docking (further referred to as 4D screening model), as implemented in ICM-Pro molecular modeling software. The 4D docking allowed to account for receptor flexibility by screening several binding pocket conformations in a single docking run. Combining of similar structural models into 4D screening models has been shown to dramatically reduce screening time, as compared to individual model screening, while maintaining most of the screening accuracy [27].

### 3.2. Prospective VLS and Hits Selections

**Figure 1:**
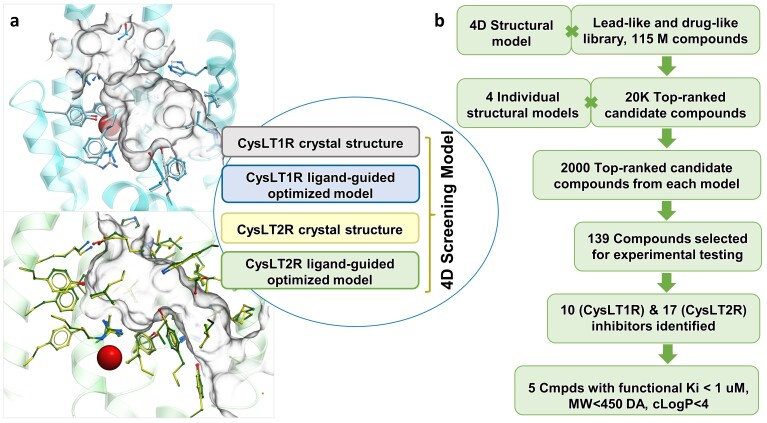
**Overview of the prospective VLS for CysLTRs.** (**a**) Optimized ligand pockets for CysLT1R (blue) and CysLT2R (green) and the composition of the 4D Screening model. Key water in the optimized pocket of CysLT1R and CysLT2R is shown by red sphere. (**b**) Flowchart of screening and ligand identification procedure.

A total of 115 million available-on-demand compounds from Enamine lead-like 350/3 and diverse drug-like libraries were employed in 4D docking into the multi-template CysLT1 and CysLT2 receptors model, as described in Methods (Figure 1). Energy-based docking was performed and a binding score for the best conformation of each ligand was evaluated. Top 20,000 molecules with the binding score better than −30 (score threshold) were selected and then redocked into individual crystal structures and LiBERO optimized models with a higher effort to ensure comprehensive conformational sampling. For each of the four individual structures, the top 2000 compounds with the highest docking score were selected for further evaluation. All molecules were clustered based on their chemical similarity and filtered to ensure chemical diversity and novelty with respect to known CysLT1R and CysLT2R ligands. A total of 155 compounds from the prospective screening were selected for experimental testing based on their binding score, predicted binding pose, and chemical diversity.

Out of the selected 155 compounds, 139 compounds (35 for CysLT1R crystal structure, 36 for CysLT1R LiBERO-optimized model, 35 for CysLT2R crystal structure, and 33 for CysLT2R LiBERO-optimized model) were synthesized and delivered by Enamine in less than 5 weeks. Enamine REAL compounds are synthesized by fast one-pot parallel synthesis, based on a highly-optimized set of reactions and qualified reagents available in stock, which assures fast delivery and high success rate (>80%), both requirements being successfully met in this study.

### 3.3. Functional Activity of Selected Compounds in IP1 Production Assay

To determine which compounds may modulate the activity of CysLT receptors, we screened the 139 predicted hits (see Supplementary Table S5) in a functional assay that measures IP1 production as an indicator of Gαq activation, which is the main signaling pathway for both receptors. An initial screen was performed in which HEK293 cells transiently expressing the wild-type receptors were directly stimulated with 30 µM compound concentrations. As expected, none of the compounds showed agonism at CysLTRs (data not shown). Then all compounds were tested for their antagonistic effect at 30 µM concentration in HEK293 cells activated with a fixed concentration of the endogenous ligand LTD4 (EC_80_; effective concentration eliciting 80% of the maximum signal or greater) (Supplementary Figures S1 and S2). Based on this screen, 10 compounds were selected as potential hits (≥50% inhibition) for CysLT1R, and 17 were selected for CysLT2R. BRI-12359 was the most effective at inhibiting LTD4-induced IP1 production, with inhibitory efficacies of 94 ± 17% and 64 ± 10% for CysLT1R and CysLT2R, respectively.

**Figure 2:**
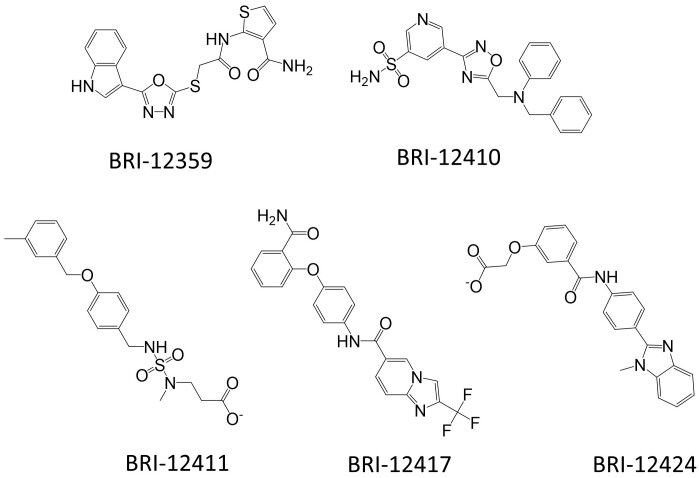
**Chemical structures of the experimentally confirmed VLS hitsfor CysLT1R and CysLT2R receptors**. All hits have Tanimoto Distances > 0.43 to known high-affinity ligands of CysLTRs (IC50 range from 0.79 to 53 nM) (see Supplementary Table S6).

**Figure 3:**
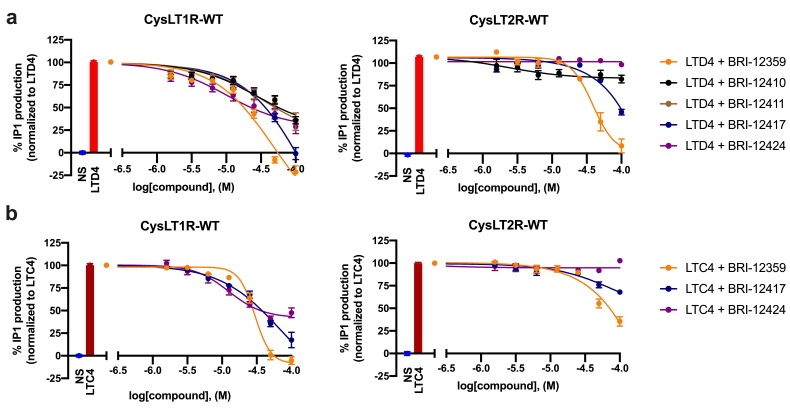
**Functional characterization of best hits in IP1 production inhibition assay.** Inhibition of LTD4- (**a**) or LTC4- (**b**) induced IP1 production by selected compounds. Experiments were performed in HEK293 cells transiently expressing the wild-type CysLT1R or CysLT2R. Curves correspond to normalized data; values from non-stimulated cells were set as 0% IP1 production and those from cells stimulated with either LTD4 or LTC4 were set as 100% IP1 production. Data represent mean ± SEM of three independent experiments, tested in quadruplicate. NS = non-stimulated.

The potencies of the best compounds at inhibiting the agonist-induced response were tested in the antagonist mode at concentrations ranging from 1.5 to 100 µM. For the CysLT1R, five hits with robust dose response were identified: BRI-12359, BRI-12410, BRI-12411, BRI-12417, and BRI-12424. Their chemical structures are provided in Figure 2. Of these, BRI-12359 and BRI-12417 were able to completely inhibit the agonist-mediated response, with BRI-12359 being the more potent of the two (Figure 3a; Table 2). As shown in Table 2, the identified hits display sub-micromolar or low micromolar K_i_ values in inhibiting LTD4-mediated IP1 production. These functional K_i_ values were calculated using the Cheng-Prusoff equation. Compounds BRI-12410, BRI-12411, and BRI-12424 showed partial antagonism with K_i_ values in the 0.2 to 0.7 µM range. For CysLT2R, only BRI-12359 fully abolished the LTD4-induced response (K_i_ = 6.46 ± 0.43 µM), while BRI-12417 displayed partial inhibition (54 ± 6%) at 100 µM (Supplementary Table S1 and Figure S3).

### 3.4. Characterization of Hits for Their Ability to Inhibit LTC4-Induced IP1 Production

Considering that LTC4 is another prominent endogenous leukotriene that activates both CysLT1R and CysLT2R, we tested the three most promising hits (BRI-12359, BRI-12417 and BRI-12424) in the IP1 assay using LTC4 as agonist. As one can see in Figure 3b, the compounds showed similar profiles with LTC4 vs LTD4 at the CysLT1R, with even slightly better sub-micromolar potencies (Ki = 0.7 ± 0.2 µM for both BRI-12417 and BRI-12359). However, BRI-12359 and BRI-12417 were less effective at inhibiting the LTC4-induced response at CysLT2R at the EC_80_ concentration of LTC4 (Table 2), and their K_i_ values could not be calculated.

### 3.5. Further Functional Characterization of Best Hits in IP1 Production Assay

**Figure 4:**
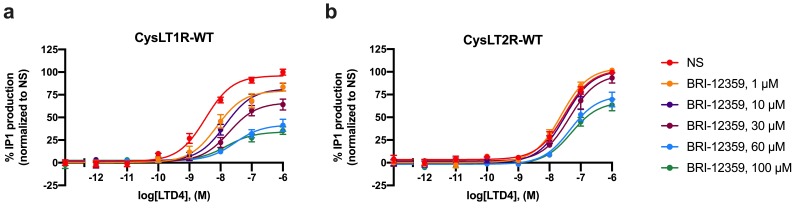
**pA2 determination of LTD4-induced IP1 production of BRI-12359.** Inhibition of LTD4-induced IP1 production by selected compounds (fixed concentration of LTD4). Experiments were performed in HEK293 cells transiently expressing the wild-type CysLT1R (**a**) or CysLT2R (**b**). Curves correspond to normalized data; values from non-stimulated cells were set as 0% IP1 production and those from cells stimulated with LTD4 were set as 100% IP1 production. Schild analysis revealed that the pA2 values for compound BRI-12359 are 6.5 ± 0.1 and 4.0 ± 0.2 (K_B_ = 0.34 ± 0.09 µM and 105 ± 37 µM) for the CysLT1R-WT and CysLT2R-WT, respectively. Data represent mean ± SEM of three independent experiments, tested in quadruplicate. NS = non-stimulated.

Recognizing that the sensitivity of the above assays depends on the concentration of agonist used, we also tested compound BRI-12359 in a more elaborate experimental paradigm designed to ascertain its pA2 values in Schild analysis as a more robust estimate of functional potency. Here, cells were stimulated with fixed concentrations of BRI-12359 before receiving a range of LTD4 concentrations (10^−12^ M to 10^−6^ M). As shown in Figure 4, BRI-12359 inhibited LTD4 signaling in a concentration-dependent manner. Schild analysis revealed that the pA2 values for BRI-12359 are 6.5 ± 0.1 and 4.0 ± 0.2 (K_B_ = 0.34 ± 0.09 µM and 105 ± 37 µM) for the CysLT1R and CysLT2R, respectively. The LTD4 EC_50_ and Emax values are provided in Table 3.

### 3.6. Inhibition of Constitutively Active CysLT2R Mutant L129Q Involved in Uveal Melanoma

**Figure 5:**
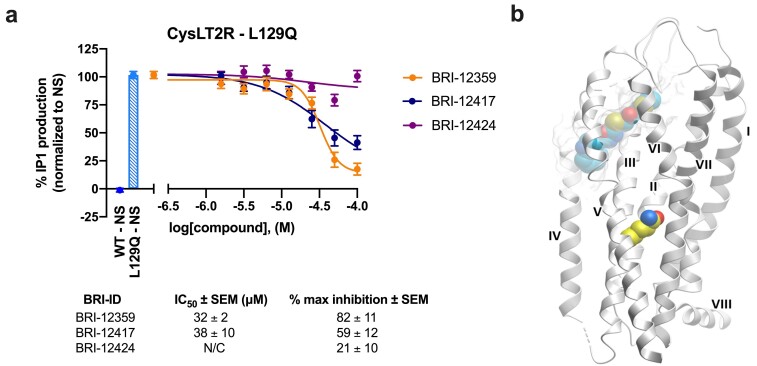
**Effect of best hits on constitutive activity of the CysLT2R mutant L129Q**. (**a**) Inhibition of IP1 accumulation in HEK293 cells transiently expressing the CysLT2R mutant L129Q. Curves correspond to normalized data: values from non-stimulated cells expressing the WT receptor were set as 0% IP1 production and those from non-stimulated cells expressing the L129Q mutant were set as 100% IP1 production. Data represent mean ± SEM of three independent experiments, tested in quadruplicate. WT = wild-type; NS = non-stimulated. (**b**) Docking of BRI-12359 to the L129Q mutant. Docking pose of BRI-12359 is shown with cyan carbon atoms representation in grey transparent binding pocket of CysLT2R receptor, and L129Q mutation shown with yellow carbon atoms in CPK presentation.

Given the efficient antagonism of the novel BRI-12359, BRI-12417 and BRI-12424 compounds, we hypothesized that these compounds might also inhibit receptor signaling at constitutively active CysLTR mutants, thereby acting as inverse agonists. This effect would be particularly interesting in the case of the L129Q CysLT2R mutant identified in uveal melanoma patients [7,10,36,37]. We tested these three compounds in the functional IP1 assay using cells transiently expressing the L129Q CysLT2R mutant, directly stimulated with a range of compound concentrations (1.6 to 100 μM). As can be seen in Figure 5**,** the IP1 accumulation caused by signaling of the constitutively active CysLT2R L129Q mutant was reduced in cells stimulated with BRI-12359 and BRI-12417. This reduction was concentration-dependent, with potencies (IC_50_) in the 30 to 40 μM range, where BRI-12359 shows practically full inhibition of the IP1 production by CysLT2R L129Q mutant at a 100 μM concentration.

### 3.7. Chemical and Conformational Diversity of Hit Compounds

**Figure 6:**
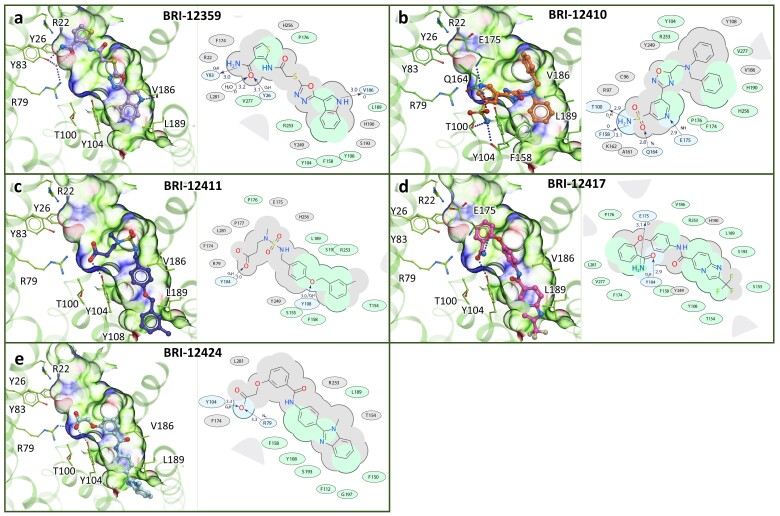
**Predicted binding posesof the experimental hits in the refined CysLT1R binding pocket.** Ligands are shown is stick presentation. Receptor is shown in cartoon and thin stick presentation with carbons colored green. The pocket is shown as transparent surface colored according to properties (green: hydrophobic, blue: H-bond donor, red: H-bond acceptor). 2D diagrams depict ligand interactions with receptor residues, with H-bonds shown as dashed lines and distances in Å.

Experimentally confirmed hits have diverse chemical structures and predicted binding poses exploring different parts of the binding pocket. Compounds BRI-12359 and BRI-12417 have the same polar amide group, but the predicted binding poses show that these two ligands have different critical interactions with the CysLT1R binding pocket (Figure 6). The amide of BRI-12359 creates H-bond with the Y83^2.64^, while amide of the BRI-12417 forms H-bond with the Y104^3.33^. Moreover, compound BRI-12359 occupies the upper part of the binding pocket, while compound BRI-12417 leaves this area empty.

BRI-12411 and BRI-12424 contain carboxy groups that interact with the R79^2.60^ and Y104^3.33^ anchor residues in the CysLT1R binding pocket. Compound BRI-12410 with the most distinct chemical structure shows unique binding pose forming H-bond with T100^3.29^ and hydrophobic interactions between the benzene ring and hydrophobic parts of the CysLT1R binding pocket.

All hits have predicted clogP values below 4 and molecular weight MW < 450 Da (see Table 2). These properties make them especially valuable as potential drug discovery leads, as CysLT1R and CysLT2R receptors are lipid receptors and tend to have highly hydrophobic ligands with a molecular weight often greater than 600 Da and cLogP > 6.

## 4. Discussion

Cysteinyl leukotriene receptors are important clinical targets for the treatment of asthma and related disorders [1,3,4,15,38], and have recently been considered for other indications related to modulation of the inflammatory response [39,40,41,42], as well as cancer [7,8,9,10,36,43]. However, the discovery of lead compounds for CysLT1R and CysLT2R receptors is challenging due to the lipophilic nature of binding pockets of these receptors, which tend to bind large hydrophobic molecules with physicochemical properties that are not favorable for drug discovery. In the present study, we employed multi-template docking of CysLT1R and CysLT2R receptors in ultra-large virtual ligand screening to find novel lead-like and drug-like compounds with potential utility in drug discovery. Multi-template 4D screening model, comprising CysLT1R and CysLT2R crystal structures and the corresponding LiBERO-optimized models, allowed to efficiently screen Enamine REAL libraries containing more than 115 million available in stock and make-on-demand compounds and find potential binders for both receptors in a single screening run. The experimental testing of computationally predicted hits identified five compounds with functional activity at CysLT1R receptor. Compound BRI-12359 showed full antagonism at both CysLT1R and CysLT2R receptors, while compounds BRI-12410, BRI-12411, BRI-12417, and BRI-12424 showed partial antagonism at CysLT1R receptor with strong selectivity.

The potencies of these compounds were verified in assays where CysLT1 was stimulated by a different endogenous agonist, LTC4, with functional affinities consistent between LTD4 and LTC4, and in the case of BRI-12359, BRI-12417, and BRI-12424 with some improvement in the K_i_ values, supporting activity of all five compounds in submicromolar range. This is also consistent with more comprehensive Schild analysis, revealing the K_B_ = 0.34 ± 0.09 mM for BRI-12359. Importantly, the predicted physicochemical properties of identified hits, such as molecular weight MW < 460 Da, hydrophobicity clogP < 3.6, and solubility logS > −4.3 (see Table 1), make them promising candidates for further lead optimization to improve their antagonistic potencies. Interestingly, all five validated hits had the molecular weight 350 < MW < 460 Da and were derived from the REAL drug-like library. This observation suggests that the large and open ligand binding pocket of CySLTRs, which combines distinct hydrophilic and hydrophobic parts, requires larger ligands for high antagonist potency. Success of previous VLS campaigns with fragment/lead subsets of REAL or other databases [18,19,20,21], however, suggests that this observation is receptor-specific, and smaller compounds may have utility for other GPCRs.

Experimental validation of the predicted hits also showed that ligand-optimized models of the receptor have a somewhat improved predictive power, with 3 out of 5 best hits (including BRI-12359) identified with the LiBERO-optimized models. This observation supports the use of ligand-optimized models when known high-affinity ligands for the target are available [28].

Since the discovery of the constitutive activation of the CysLT2R-L129Q mutant in uveal melanoma, inhibition of the receptor has been considered as a potential treatment for this devastating disease. The inverse agonism shown in this study for BRI-12359 suggests that new lead chemotypes of such inhibitors can be discovered via structure-based VLS, and suggests a possibility of optimizing these scaffolds.

## 5. Conclusions

Screening of the ultra-large library of tangible Enamine compounds for CysLT1R and CysLT2R ligands yielded the discovery of five new sub-micromolar antagonists. The most potent full antagonist, BRI-12359, demonstrated dual antagonism with CysLT1R preference (K_B_ = 340 nM vs 105 μM), while other compounds showed strong CysLT1R selectivity. Unlike previous drugs and drug candidates for CysLTRs, these new chemotypes are small (360 < MW < 460 Da) and hydrophilic (logP < 4), which makes them potential candidates for lead optimization with new drug-like properties. Moreover, we showed that almost full inhibition of the constitutively active mutant L129Q is possible with BRI-12359. Inhibitors of this mutant receptor activity may be further developed into a potential treatment of uveal melanoma.

## Figures and Tables

**Table 1:** Performance evaluation for CysLT1R and CysLT2R crystal structures and LiBERO-optimized models.

	Number of Water Molecules	ROC	NSA	Score Average	Best Score
CyLT1, crystal structure	0	80	60	−37	−41
CyLT1, LiBERO structure	1	86	74	−46	−52
CyLT2, crystal structure	1	87	77	−34	−37
CyLT2, LiBERO structure	1	89	82	−39	−43
4D screening model (combined)	as above	90	82	−45	−50

**Table 2:** Experimentally confirmed hits for CysLT1 and CysLT2 receptors.

	CysLT1R (LTD4)		CysLT2R (LTD4)		CysLT1R (LTC4)		CysLT2R (LTC4)		MW, Da	cLogP	cLogS	Tanimoto Distance
BRI-ID	Ki ± SEM μM	% Max Inhibition ± SEM	Ki ± SEM μM	% Max Inhibition ± SEM	Ki ± SEM μM	% Max Inhibition ± SEM	Ki ± SEM μM	% Max Inhibition ± SEM				
BRI-12359	1.03 ± 0.28	121 ± 13	6.46 ± 0.43	92 ± 15	0.72 ± 0.26	105 ± 7	N/C	65 ± 11	399	1.59	−2.25	0.57
BRI-12410	0.59 ± 0.12	64 ± 7	N/C	18 ± 9					421	3.29	−3.43	0.62
BRI-12411	0.72 ± 0.16	66 ± 18							392	3.48	−3.64	0.54
BRI-12417	3.1 ± 0.63	100 ± 17	N/C	54 ± 6	0.68 ± 0.12	83 ± 14	N/C	32 ± 5	440	3.69	−4.29	0.54
BRI-12424	0.22 ± 0.03	71 ± 19	N/C	7 ± 5	0.18 ± 0.03	52 ± 9	N/C	9 ± 5	400	3.32	−3.71	0.43

N/C—not converged; shaded cells—not tested.

**Table 3:** Inhibition of LTD4-induced IP1 production by BRI-12359.

	CysLT1R-WT			CysLT2R-WT		
[BRI-12359] μM	LTD4 EC_50_ ± SEM nM	LTD4 Emax ± SEM%	pA2 ± SEM −log	LTD4 EC_50_ ± SEM nM	LTD4 Emax ± SEM %	pA2 ± SEM −log
0	3.3 ± 0.3	100 ± 6	6.5 ± 0.1	30 ± 3	100 ± 3	4.0 ± 0.2
1	8 ± 1	84 ± 9		24 ± 2	101 ± 3	
10	14 ± 2	83 ± 9		31 ± 3	99 ± 3	
30	19 ± 4	64 ± 12		43 ± 6	93 ± 10	
60	29 ± 8	41 ± 13		45 ± 8	70 ± 16	
100	15 ± 4	35 ± 8		50 ± 8	64 ± 13	

## Supplementary Materials

The following are available online at https://www.mdpi.com/2218-273X/10/12/1634/s1, Figure S1: Evaluation of CysLT1R and CysLT2R structures and ligand-guided optimized models performance in benchmark docking, Figure S2: Functional screening of selected compounds at CysLT1R receptor in IP1 production assay, Figure S3: Functional screening of selected compounds at CysLT2R receptor in IP1 production assay, Figure S4: Functional characterization of selected compounds in IP1 production assay. Table S1: Efficacy of selected compounds in functional IP1 assay, Table S2: CysLT1R active compounds used in ligand-guided optimization algorithm. Table S3: CysLT2R active compounds used in ligand-guided optimization algorithm, Table S4. Decoy compounds used in ligand-guided optimization algorithm, Table S5: Experimentally tested VLS hit compounds. Experimentally confirmed hits for CysLT1R are in bold, for CysLT2R are in italic, Table S6: Chemical similarity between experimental hits and known CysLTR ligands.
