# Supplementary material for: Structure-Based Virtual Screening of Ultra-Large Library Yields Potent Antagonists for a Lipid GPCR
Source: Biomolecules. 2020 Dec 3;10(12):1634. doi: 10.3390/biom10121634 (PMC7761830; doi:10.3390/biom10121634)
Supplement: Supplementary file 1 [file biomolecules-10-01634-s001.zip › Supplementary_Information.docx]

**SUPPLEMENTARY INFORMATION**

***
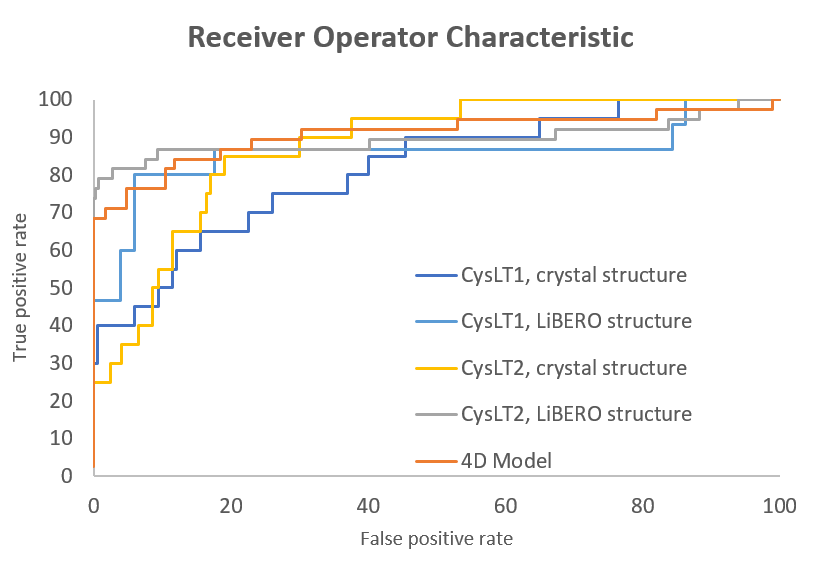
***

***Supplementary Figure S1: Evaluation of CysLT1R and CysLT2R structures and ligand-guided optimized models performance in benchmark docking.***


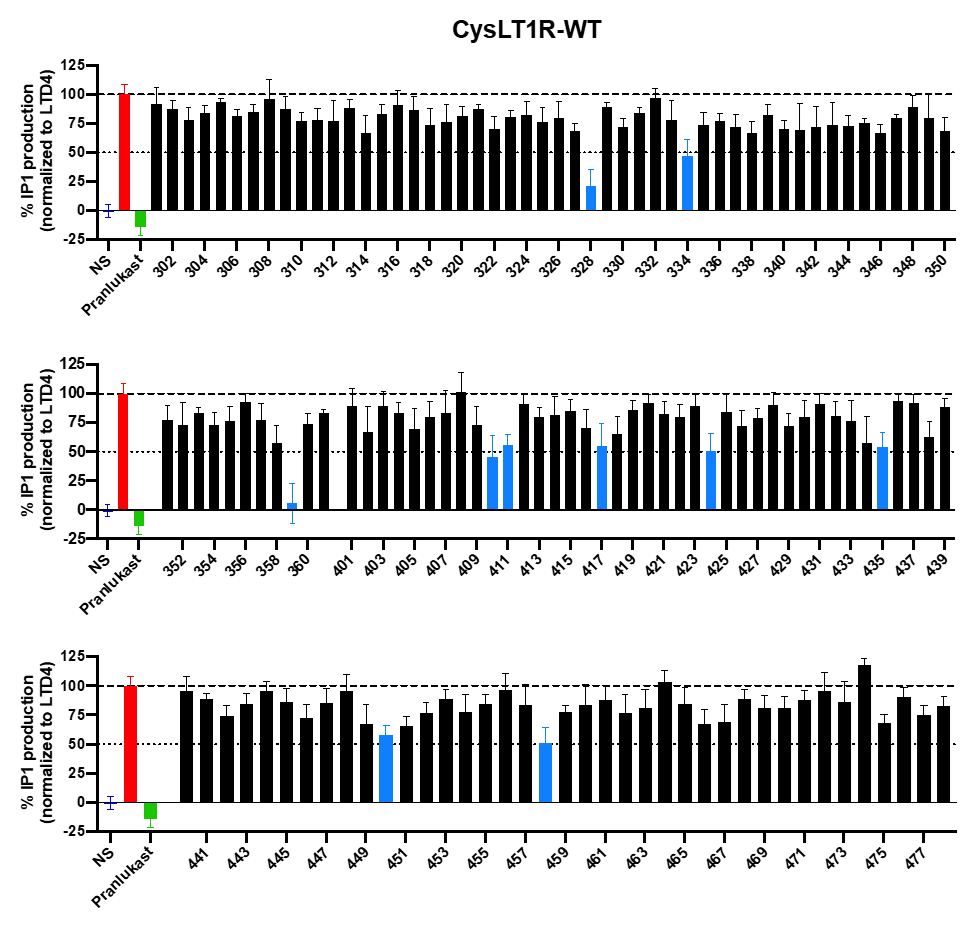


***Supplementary Figure S2: Functional screening of selected compounds at CysLT1R receptor in IP1 production assay.*** Inhibition of LTD4-induced IP1 production by selected compounds (30 μM) in HEK293 cells transiently expressing the wild-type CysLT1R receptor. Data sets were normalized; values from non-stimulated cells were set as 0% IP1 production and those from cells stimulated with either LTD4 were set as 100 % IP1 production. Data represent mean ± SEM of at least two independent experiments, tested in quadruplicate. For clarity’s sake, compounds are identified here by the last three digits of the BRI-ID only. Compounds presented in light blue were selected for further study. NS = non-stimulated.

***
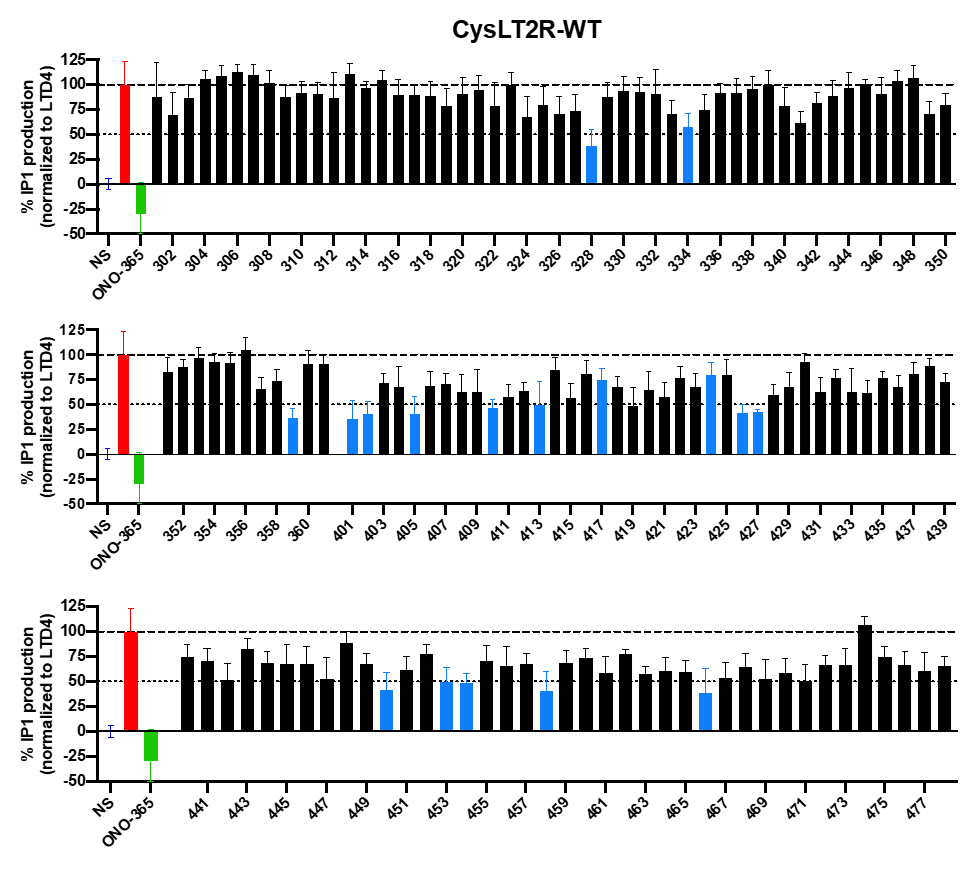
***

***Supplementary Figure S3: Functional screening of selected compounds at CysLT2R receptor in IP1 production assay.*** Inhibition of LTD4-induced IP1 production by selected compounds (30 μM) in HEK293 cells transiently expressing the wild-type CysLT2R receptor. Data sets were normalized; values from non-stimulated cells were set as 0% IP1 production and those from cells stimulated with either LTD4 were set as 100 % IP1 production. Data represent mean ± SEM of at least two independent experiments, tested in quadruplicate. For clarity’s sake, compounds are identified here by the last three digits of the BRI-ID only. Compounds presented in light blue were selected for further study. Note that compounds BRI-12417 and BRI-12424 were selected despite a max inhibition < 50 %, as they were found to be interesting hits for CysLT1R. NS = non-stimulated.

**
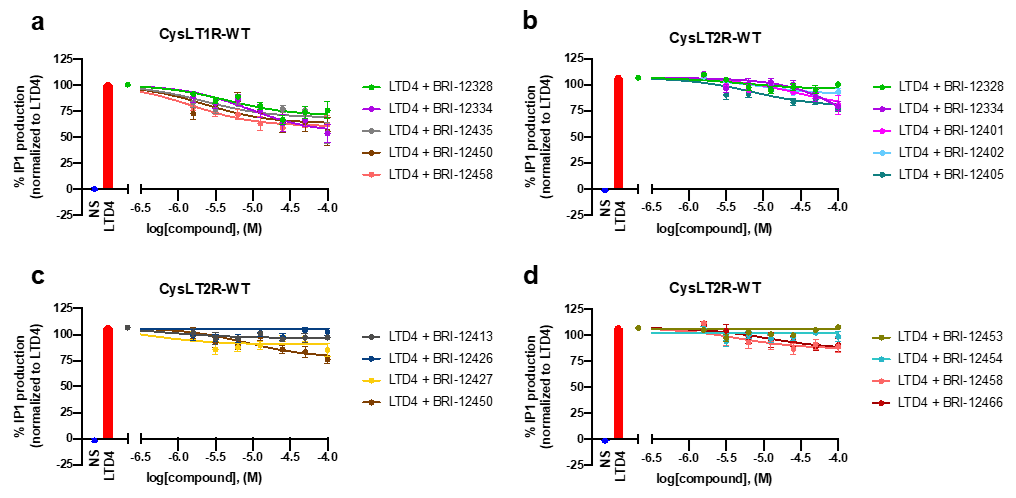
**

***Supplementary Figure S4: Functional characterization of selected compounds in IP1 production assay.*** Inhibition of LTD4-induced IP1 production by selected compounds (fixed EC_80_ concentration of LTD4) in HEK293 cells transiently expressing the wild-type CysLT1R (**a**) or CysLT2R receptor (**b-d**). Curves correspond to normalized data; values from non-stimulated cells were set as 0% IP1 production and those from cells stimulated with LTD4 were set as 100 % IP1 production. Data represent mean ± SEM of three independent experiments, tested in quadruplicate. NS = non-stimulated.

***Supplementary Table S1: Efficacy of selected compounds in functional IP1 assay***

|  | **CysLT1R-WT** | **CysLT2R-WT** |
| --- | --- | --- |
| **BRI-ID** | **% max inhibition ± SEM** | **% max inhibition ± SEM** |
| BRI-12328 | 33 ± 8 | 5 ± 7 |
| BRI-12334 | 46 ± 15 | 21 ± 8 |
| BRI-12401 |  | 19 ± 15 |
| BRI-12402 |  | 8 ± 4 |
| BRI-12405 |  | 23 ± 6 |
| BRI-12413 |  | 5 ± 6 |
| BRI-12426 |  | 5 ± 9 |
| BRI-12427 |  | 15 ± 13 |
| BRI-12435 | 46 ± 20 |  |
| BRI-12450 | 44 ± 25 | 24 ± 8 |
| BRI-12453 |  | 3 ± 7 |
| BRI-12454 |  | 6 ± 10 |
| BRI-12458 | 38 ± 16 | 13 ± 10 |
| BRI-12466 |  | 12 ± 6 |

*Shaded cells – not tested

***Supplementary table S2. CysLT1R active compounds used in ligand-guided optimization algorithm*.**

| **ChEMBL_ID** | **SMILES** | **MW** | **pAct** |
| --- | --- | --- | --- |
| CHEMBL603 | Cc1ccccc1S(NC(c1ccc(Cc2cn(C)c3ccc(cc23)NC(=O)OC2CCCC2)c(c1)OC)=O)(=O)=O | 575.2 | 11.4 |
| CHEMBL344180 | CC(C)(c1ccccc1CC[C@H](c1cccc(C=Cc2ccc3CCCCc3n2)c1)SCC1(CC1)CC(O)=O)O | 555.3 | 9.7 |
| CHEMBL139757 | CC(C)(c1ccccc1CC[C@H](c1cccc(C=Cc2ccc3CSCc3n2)c1)SCC1(CC1)CC(O)=O)O | 559.2 | 9.7 |
| CHEMBL3597618 | Cc1c(CCCCOc2ccc(C#Cc3cccc4c(CCCC(O)=O)c(C)n(CC(O)=O)c34)cc2)cccc1[Cl] | 571.2 | 9.7 |
| CHEMBL288943 | CCCCCC=CCC=CC=CC=C[C@H]([C@H](CCCC(O)=O)O)SC[C@@H](C(NCC(O)=O)=O)N | 496.3 | 9.5 |
| CHEMBL313187 | CC(C)(c1ccc(CC[C@H](c2cccc(C=Cc3ccc4c(c(c(s4)[Cl])[Cl])n3)c2)SCC2(CC2)CC(O)=O)cc1)O | 625.1 | 9.4 |
| CHEMBL3403186 | C(CCOc1ccc(C=Cc2cccc3c2OC(CN3CCCC(O)=O)c2nnn[nH]2)cc1)Cc1ccccc1 | 539.3 | 9.2 |
| CHEMBL21333 | C(CCOc1ccc(cc1)C(Nc1cccc2C(C=C(c3nn[nH]n3)Oc12)=O)=O)Cc1ccccc1 | 481.2 | 9.1 |
| CHEMBL341547 | CCCCc1c(C)ccc(C=Cc2cccc(c2)[C@@H](CCc2ccccc2C(C)(C)O)SCC2(CC2)CC(O)=O)n1 | 571.3 | 9.0 |
| CHEMBL131611 | C(CC(O)=O)Cc1ccc(cc1)NC(c1ccc2ccc(cc2c1)OCc1ccc2ccccc2n1)=O | 490.2 | 8.8 |
| CHEMBL1214477 | CC1(C)[C@@H](Cc2cc(ccc2OC)NS(C(F)(F)F)(=O)=O)[C@@H](CO)c2cc(ccc2O1)OCc1nc2cc(c(cc2s1)F)F | 658.1 | 8.7 |
| CHEMBL130846 | C(COCc1ccc2ccc(cc2c1)OCc1ccc2ccccc2n1)c1ccc(cc1)C(O)=O | 463.2 | 8.6 |
| CHEMBL140058 | CC(C)(c1ccccc1CC[C@H](c1cccc(C=Cc2ccc3c(CCS3)n2)c1)SCC1(CC1)CC(O)=O)O | 559.2 | 8.5 |
| CHEMBL89340 | CN(C)C(CCS[C@@H](c1cccc(C=Cc2ccc3ccc(cc3n2)[Cl])c1)SCCC(O)=O)=O.[Na] | 537.1 | 8.5 |
| CHEMBL423333 | C(CNC(c1ccc2ccc(cc2c1)OCc1ccc2ccccc2n1)=O)c1ccc(cc1)c1nn[nH]n1 | 500.2 | 8.5 |
| CHEMBL128641 | CC(C)(CNS(c1ccc2ccc(cc2c1)OCc1ccc2ccccc2n1)(=O)=O)c1ccc(cc1)C(O)=O | 540.2 | 8.4 |
| CHEMBL1214531 | Cc1ccc2ccc(COc3ccc4c(c3)[C@H](CO)[C@H](Cc3cc(ccc3OC)NS(C(F)(F)F)(=O)=O)C(C)(C)O4)nc2c1 | 630.2 | 8.4 |
| CHEMBL3810210 | COc1cccc2c(C=CC(Nc3cccc(C=Cc4ccc5ccc(cc5n4)[Cl])c3)=O)c(C(O)=O)[nH]c12 | 523.1 | 8.2 |
| CHEMBL11905 | C(=Cc1cccc(C=Cc2ccc3ccccc3n2)c1)C(c1cc(cc(C(O)=O)c1O)[Cl])=O | 455.1 | 8.2 |
| CHEMBL132167 | C(c1ccc(cc1)C(O)=O)OC(Nc1ccc2ccc(cc2c1)OCc1ccc2ccccc2n1)=O | 478.2 | 8.0 |
| CHEMBL97356 | CCCC(CCc1nnn[nH]1)Cc1ccc(cc1)OCc1ccc2ccccc2n1 | 401.2 | 8.0 |
| CHEMBL416769 | C(=Cc1ccc2ccccc2n1)c1cccc(c1)C1=CC(c2cc(cc(C(O)=O)c2O1)[Cl])=O | 453.1 | 8.0 |

***Supplementary table S3. CysLT2R active compounds used in ligand-guided optimization algorithm.***

| **ChEMBL_ID** | **SMILES** | **MW** | **pAct** |
| --- | --- | --- | --- |
| CHEMBL3403187 | C(CC(O)=O)CN1CC(C(O)=O)Oc2c(C=Cc3ccc(cc3)OCCCCOc3ccccc3)cccc12 | 531.2 | 9.5 |
| CHEMBL3401689 | C(CCOc1ccc(cc1)C(Nc1cccc2c1OC(CN2CCCC(O)=O)c1nn[nH]n1)=O)Cc1ccccc1 | 556.2 | 9.3 |
| CHEMBL3342959 | C(CCc1ccccc1)CCOc1ccc(cc1)C(Nc1cccc2c1OC(CN2CCCC(O)=O)C(O)=O)=O | 546.2 | 9.2 |
| CHEMBL3342964 | C(CCOc1ccc(cc1)C(Nc1cccc2c1O[C@@H](CN2CCCC(O)=O)C(O)=O)=O)Cc1ccccc1 | 532.2 | 9.1 |
| CHEMBL3597618 | Cc1c(CCCCOc2ccc(C#Cc3cccc4c(CCCC(O)=O)c(C)n(CC(O)=O)c34)cc2)cccc1[Cl] | 571.2 | 9.1 |
| CHEMBL3597616 | Cc1c(CCCCOc2ccc(C#Cc3cccc4c(CC(O)=O)c(C)n(CC(O)=O)c34)cc2)cccc1[Cl] | 543.2 | 9.1 |
| CHEMBL3597530 | Cc1c(CCCC(O)=O)c2cccc(C=Cc3ccc(cc3)OCCCCc3c(ccc(c3F)F)F)c2n1CC(O)=O | 579.2 | 9.0 |
| CHEMBL3597532 | Cc1c(CCCC(O)=O)c2cccc(C=Cc3ccc(cc3)OCCCCc3cc(c(c(c3F)F)F)F)c2n1CC(O)=O | 597.2 | 8.8 |
| CHEMBL3597525 | C(CCOc1ccc(C=Cc2cccc3c(CCCC(O)=O)cn(CC(O)=O)c23)cc1)Cc1c(ccc(c1F)F)F | 565.2 | 8.7 |
| CHEMBL3403186 | C(CCOc1ccc(C=Cc2cccc3c2OC(CN3CCCC(O)=O)c2nn[nH]n2)cc1)Cc1ccccc1 | 539.3 | 8.5 |
| CHEMBL3597631 | Cc1c(CCCC(O)=O)c2cccc(C#Cc3ccc(cc3)OCCCCc3c(cc(c(c3F)F)F)F)c2n1CCCC(O)=O | 623.2 | 8.4 |
| CHEMBL3342944 | C1CCC(CC1)OCCCCOc1ccc(CCCOc2ccc(cc2C(NC2CCCC(C2)C(O)=O)=O)C(O)=O)cc1 | 595.3 | 8.4 |
| CHEMBL3597527 | Cc1c(CCCCOc2ccc(C=Cc3cccc4c(CCCC(O)=O)cn(CC(O)=O)c34)cc2)cccc1[Cl] | 559.2 | 8.3 |
| CHEMBL3597528 | Cc1c(CCCC(O)=O)c2cccc(C=Cc3ccc(cc3)OCCCCc3ccccc3)c2n1CC(O)=O | 525.3 | 8.3 |
| CHEMBL3597535 | Cc1c(CCCCOc2ccc(C=Cc3cccc4c(CCCC(O)=O)c(C)n(CC(O)=O)c34)cc2)cccc1[Cl] | 573.2 | 8.3 |
| CHEMBL3597624 | Cc1c(CCCCOc2ccc(C#Cc3cccc4c(CCCC(O)=O)c(C)n(CCCC(O)=O)c34)cc2)cccc1[Cl] | 599.2 | 8.3 |
| CHEMBL3401687 | C(CCOc1ccc(C=Cc2cccc3c2OC(CN3CCCC(O)=O)C(O)=O)cc1)Cc1ccccc1 | 515.2 | 8.2 |
| CHEMBL3401691 | C(CCOc1ccc(cc1)C(Nc1cccc2c1OC(CN2CCCC(O)=O)C(NS(c1ccccc1)(=O)=O)=O)=O)Cc1ccccc1 | 671.2 | 8.2 |
| CHEMBL3597617 | Cc1c(CCCCOc2ccc(C#Cc3cccc4c(CCC(O)=O)c(C)n(CC(O)=O)c34)cc2)cccc1[Cl] | 557.2 | 8.2 |
| CHEMBL3597522 | C(CC(O)=O)Cc1cn(CC(O)=O)c2c(C=Cc3ccc(cc3)OCCCCOc3ccccc3)cccc12 | 527.2 | 8.2 |
| CHEMBL3597534 | Cc1c(CCCCOc2ccc(C=Cc3cccc4c(CCCC(O)=O)c(C)n(CC(O)=O)c34)cc2)cccc1F | 557.3 | 8.1 |
| CHEMBL3597633 | Cc1c(CCCCOc2ccc(C#Cc3cccc4c(CCCC(O)=O)c(C)n(CCCC(O)=O)c34)cc2)cccc1F | 583.3 | 8.1 |

***Supplementary*** ***table S4. Decoy compounds used in ligand-guided optimization algorithm*.**

| **ID** | **SMILES** | **MW** |
| --- | --- | --- |
| CHEMBL11521 | C1=C(c2ccc3ccccc3n2)Oc2c(cc(cc2C1=O)[Br])C(O)=O | 395.0 |
| CHEMBL11778 | C(c1ccc2ccccc2c1)Oc1cccc(C=CC(c2cc(ccc2O)c2nn[nH]n2)=O)c1 | 448.2 |
| CHEMBL1213781 | CN(C)CCOC1=C(c2ccc(cc2)OCc2ccc3ccccc3n2)Oc2c(cccc2C1=O)C(O)=O | 510.2 |
| CHEMBL1213782 | C1CC=C(COc2ccc(C=CC(c3cc(ccc3O)C(O)=O)=O)cc2)C=C1 | 376.1 |
| CHEMBL1213844 | C1=C(c2ccc(cc2)Oc2ccccc2)Oc2c(cccc2C1=O)C(O)=O | 358.1 |
| CHEMBL1213846 | C(CCOc1ccc(cc1)[Cl])COc1ccc(C=CC(c2ccccc2OCC(O)=O)=O)cc1 | 480.1 |
| CHEMBL1213918 | C(CCOc1ccc(C=CC(c2cc(ccc2OCC#N)C(O)=O)=O)cc1)COc1ccccc1 | 471.2 |
| CHEMBL1214416 | C(c1nc2ccccc2[nH]1)Oc1ccc(C=CC(c2cc(cc(C(O)=O)c2O)[Cl])=O)cc1 | 448.1 |
| CHEMBL1289626 | C(CCN1CCc2cc(ccc2[C@H](C1)O)O)Cc1ccccc1 | 311.2 |
| CHEMBL161502 | CCCCCCCc1ccc(CC=C[C@H]([C@H](CCCC(O)=O)O)SCc2ccc(cc2N)C(O)=O)cc1 | 527.3 |
| CHEMBL1650844 | [H][C@]12CNC[C@]2([H])CN(C1)c1nc2ccccc2n1C1CCN(CC1)C1(CCCCCCC1)CO | 451.3 |
| CHEMBL174471 | CCCCCc1ccc(C=CC(Nc2ccccc2OCC(O)=O)=O)cc1 | 367.2 |
| CHEMBL18140 | C(c1ccc2ccccc2n1)Oc1cccc(c1)NS(CC(F)(F)F)(=O)=O | 396.1 |
| CHEMBL18402 | C(CCOc1ccc(cc1)c1nnn(CCCCc2nnn[nH]2)n1)Cc1ccccc1 | 418.2 |
| CHEMBL18450 | C(Cc1nnn[nH]1)COc1ccccc1OCc1ccc2ccccc2n1 | 361.2 |
| CHEMBL18750 | C(c1cccc(c1)C(O)=O)n1nc(c2cccc(CSc3ccc4ccccc4n3)c2)nn1 | 453.1 |
| CHEMBL1885748 | C(c1cc(cc(c1)[Cl])[Cl])Nc1c(cncn1)c1ccc2c(c1)OCO2 | 373.0 |
| CHEMBL19304 | C(c1ccccc1C(O)=O)n1nc(c2cccc(c2)OCc2nc3ccccc3s2)nn1 | 443.1 |
| CHEMBL217053 | C(C(O)=O)Oc1ccc(cc1C(c1cnn(c1)c1ccccc1)=O)[Br] | 400.0 |
| CHEMBL22016 | CCCc1c(ccc(C(C)=O)c1O)OCCCCCOc1cc2c(CC[C@H](C(O)=O)O2)cc1C(C)=O | 498.2 |
| CHEMBL2413269 | CCCCCCCCOc1ccc(C=CC(c2ccc(cc2OCC(O)=O)C(O)=O)=O)cc1 | 454.2 |
| CHEMBL2440659 | Cc1csc(NC(c2cc(cc(c2)F)Oc2cncnc2)=O)n1 | 330.1 |
| CHEMBL2442750 | Cc1c(CC(O)=O)c2cccnc2n1Cc1ccc(cc1)S(C)(=O)=O | 358.1 |
| CHEMBL277748 | C(c1ccc2ccccc2c1)Oc1cccc(c1)NS(C(F)(F)F)(=O)=O | 381.1 |
| CHEMBL285175 | COC(CCC(Nc1cccc(COc2ccc3ccccc3c2)c1)=O)=O | 363.1 |
| CHEMBL3099899 | COc1ccc(CNC(c2cc(cnc2c2cccnc2)c2cc(cc(c2)[Cl])[Cl])=O)cc1OC | 493.1 |
| CHEMBL3262876 | C(C(N1CCN(CC1)C(COc1ccc2ccccc2c1)=O)=O)c1ccccc1 | 388.2 |
| CHEMBL3262896 | COc1ccc(cc1)C(N1CCN(CC1)C(C=Cc1ccc(cc1)[Br])=O)=O | 428.1 |
| CHEMBL3310802 | CCOC(N1CCC(CC1)CN1CCC2(CC1)CN(C(N(C)C)=O)c1c2cccn1)=O | 429.3 |
| CHEMBL3342946 | CCN(CC)C(C=C(C)c1ccc2c(c1)c(c(C(c1ccc(C#N)cc1)=O)o2)NC(C(C#N)=C(C)O)=O)=O | 510.2 |
| CHEMBL3342948 | C(CCOc1ccc(cc1)C(Nc1ccc2c(c1)O[C@@H](CN2)C(O)=O)=O)Cc1ccccc1 | 446.2 |
| CHEMBL3401692 | C(CCOc1ccc(cc1)C(Nc1cccc2c1OCCN2CCCC(O)=O)=O)Cc1ccccc1 | 488.2 |
| CHEMBL34611 | C(CCOc1ccc(CC=C[C@@H](CCC(O)=O)Sc2ccc(cc2)C(O)=O)cc1)COc1ccccc1 | 520.2 |
| CHEMBL3633184 | [H][C@]12CC[C@]([H])(C[C@H](C1)CN1CC[C@H](C1)N1Cc3ccccc3NC1=O)N2C(=O)OCC | 412.2 |
| CHEMBL36344 | CCCCCCCCCCCOc1ccc(cc1)C(SCCC(O)=O)SCCC(O)=O | 470.2 |
| CHEMBL3634745 | C(c1ccc2ccccc2n1)Oc1ccc(cc1)C1C=CC(NC=1c1ccc(cc1)F)=O | 422.1 |
| CHEMBL3763166 | C1C(Nc2ccc(cc12)S(NNc1ccc(cc1)[Br])(=O)=O)=O | 381.0 |
| CHEMBL3774784 | CCCCCCNC(=O)Oc1cccc(c1)c1ccc(cc1F)[C@H](C)C(O)=O | 387.2 |
| CHEMBL3809809 | C(=Cc1c2c(cc(cc2[nH]c1C(O)=O)[Cl])[Cl])C(=O)Oc1ccccc1 | 375.0 |
| CHEMBL3810142 | C(c1cccc(C=Cc2ccc3ccc(cc3n2)[Cl])c1)OC(C=Cc1c[nH]c2cc(cc(c12)[Cl])[Cl])=O | 532.1 |
| CHEMBL3810209 | C(Cc1ccccc1)COC(C=Cc1c2c(cc(cc2[nH]c1C(O)=O)[Cl])[Cl])=O | 417.1 |
| CHEMBL3810333 | C(CCOC(C=Cc1c2c(cc(cc2[nH]c1C(O)=O)[Cl])[Cl])=O)CC#N | 380.0 |
| CHEMBL4160748 | Cc1cc(C(F)(F)F)n2c(c(c3ccc(c(c3F)F)OC)c(C)n2)n1 | 357.1 |
| CHEMBL4172540 | C[C@@H](COC1C=CN(C(C=1)=O)c1ccc(cc1)F)Oc1ccccc1 | 339.1 |
| CHEMBL431161 | Cc1ccc(CC(Nc2ccc3ccn(Cc4ccc(cc4OC)C(O)=O)c3c2)=O)cc1 | 428.2 |
| CHEMBL50779 | CCCCCC(Nc1ccc2c(c1)cn(Cc1ccccc1C(O)=O)n2)=O | 365.2 |
| CHEMBL521179 | CN(C)CCOc1cc(ccc1NC([C@@H]1COc2ccccc2O1)=O)c1cn[nH]c1 | 408.2 |
| CHEMBL54720 | C1CN(CCN1Cc1ccccc1)c1c2cccn2c2c(ccs2)n1 | 348.1 |
| CHEMBL94421 | CCCc1c(COc2ccc3C([C@H](CCc3c2)CC(NS(c2ccccc2C)(=O)=O)=O)=O)ccc(C(C)=O)c1O | 563.2 |
| CHEMBL96207 | CCCc1c(COc2ccc(cc2)C(CCCc2nnn[nH]2)=O)ccc(C(C)=O)c1O | 422.2 |
| ZINC00834065 | CCCc1c(Cc2ccc(cc2)c2ccccc2c2nnn[nH]2)c(C(O)=O)n(CC(F)(F)F)n1 | 470.2 |
| ZINC00878852 | Cc1cc2C=C(CN(C3CCCCC3)C(NCCc3ccc(c(c3)OC)OC)=S)C(Nc2cc1C)=O | 507.3 |
| ZINC01046823 | Cc1c(cc(c2ccccc2)n1CCSc1cc(C(F)(F)F)nc2ccccc12)C(O)=O | 456.1 |
| ZINC01269758 | CCN(CC)c1ccc(cc1)[C@H]1[C@H]2C(C[C@@H](CC2=O)c2ccc(cc2)F)=Nc2ccccc2N1C(CCC(O)=O)=O | 555.3 |
| ZINC01493543 | Cn1c2cc(ccc2nc1COc1ccc(C[C@H](C(O)=O)Nc2ccccc2C(c2ccccc2)=O)cc1)OC | 535.2 |
| ZINC01533884 | CCCc1nc(c(C(O)=O)n1Cc1ccc(cc1)c1ccccc1c1nnn[nH]1)n1cccc1C(C(F)(F)F)=O | 549.2 |
| ZINC01566689 | C(c1ccccc1)N(Cc1ccccc1)C(c1c(N)nc(SCc2ccccc2)s1)=O | 445.1 |
| ZINC01829327 | CC1(C)[C@@]2(C)CC[C@@]1(C(C2=O)=O)C(NC1CCCCC1)=O | 291.2 |
| ZINC01867763 | COc1ccc(cc1)N1C(=NC2=C(C1=O)C1(CCCCC1)Cc1ccccc12)SCC(N1CCCCC1)=O | 529.2 |
| ZINC02004074 | CCCCc1nnc(n1Cc1ccc(cc1)c1ccccc1c1nnn[nH]1)S(Cc1ccc(cc1)[Cl])=O | 531.2 |
| ZINC02055960 | CCOC(C1=C(c2ccccc2)SC(=C2C(C(C)(C)N(C(c3ccccc3)=O)c3ccccc23)=S)S1)=O | 543.1 |
| ZINC02100904 | CC1=C(CCC(N[C@@H](CSCc2ccccc2)C(O)=O)=O)C(=O)Oc2cc3c(cc12)c(C)co3 | 479.1 |
| ZINC02127779 | CCCOc1ccccc1C1C2=C(CC(C)(C)CC2=O)N(CCC(O)=O)C2CC(C)(C)CC(C1=2)=O | 479.3 |
| ZINC02145319 | CCOc1ccc(cc1)n1nc2cc(C)c(cc2n1)NC(NC(c1ccc(c2ccc(cc2)[Br])o1)=O)=S | 575.1 |
| ZINC02425356 | COCCCNc1c(NS(c2cc(ccc2[Cl])[Cl])(=O)=O)nc2ccccc2n1 | 440.0 |
| ZINC02505559 | COc1ccc(C[C@H](CNC(=O)OC[C@H]2c3ccccc3c3ccccc23)C(O)=O)cc1 | 431.2 |
| ZINC02791705 | CC(C)(C)c1ccc(cc1)OCc1cc(C=Nn2c(C(F)F)nnc2S)ccc1OC | 446.2 |
| ZINC02867357 | CCCCn1c(C)c(C(C)=O)c2c(c(c(c(c12)[Cl])[Cl])[Cl])OCC(O)=O | 405.0 |
| ZINC02991189 | CCOc1ccc(cc1)OCCCCOc1cccc(c1)[Cl] | 320.1 |
| ZINC03010312 | CCOc1cc2C(N(Cc3ccc(cc3)C(NCCCOC)=O)C(=Nc2cc1OCC)S)=O | 471.2 |
| ZINC03063353 | Cc1cccc(CN2C(C(=Cc3ccc(cc3)C(N(C)Cc3ccccc3)=O)Sc3ccccc23)=O)c1 | 504.2 |
| ZINC03092952 | CCOP([C@](C(=O)OC)(C(F)(F)F)NS(c1ccccc1)(=O)=O)(=O)OCC | 433.1 |
| ZINC03108836 | c1cc(ccc1N(=O)=O)S(c1cnc(N=C(C(C(C(F)(F)F)(F)F)(F)F)O)s1)(=O)=O | 481.0 |
| ZINC03272701 | CC(C)N(c1ccccc1)c1ccc(cc1)NC(CN1C(c2ccccc2C(C(O)=O)=N1)=O)=O | 456.2 |
| ZINC03644535 | Cc1cc(c2c(c1)c(c1ccccc1)c(C(NN=Cc1ccccc1C(O)=O)=O)[nH]2)N(=O)=O | 442.1 |
| ZINC03834054 | CCCc1c(ccc2c(C(F)(F)F)noc12)OCCCSc1ccc(CC(O)=O)cc1[Cl] | 487.1 |
| ZINC03984364 | COC1(C(C(=C([C@@]1(CC=C)[Cl])O)[Cl])=NC[C@@H](COc1ccccc1)O)OC | 415.1 |
| ZINC04342633 | CCOC(C1=C(C[Br])NC(N[C@H]1c1cc(ccc1[Br])OC)=O)=O | 445.9 |
| ZINC04522146 | CC[C@H](C)[C@@H](C(Nc1ccc(cc1)S(Nc1cc(C)nc(C)n1)(=O)=O)=O)NC(c1ccccc1)=O | 495.2 |
| ZINC04618687 | COc1cc(C=Nc2ccc(cc2)N2CCOCC2)ccc1OCc1cccc(c1)C(O)=O | 446.2 |
| ZINC04802495 | CCCCCC1=CC(C=C([C@@]12C(=O)Oc1cc(c(C(O)=O)c(CCCCC)c12)O)OC)=O | 442.2 |
| ZINC04968372 | C1CCc2c(C1)c1cc(ccc1nc2SCC(N)=O)F | 290.1 |
| ZINC05344912 | Cc1ccc(C)c(c1)NS(c1cc2C(c3cc(cc(c3c2c(c1)N(=O)=O)N(=O)=O)S(Nc1cc(C)ccc1C)(=O)=O)=O)(=O)=O | 636.1 |
| ZINC05922909 | C#CC=CCCCCC=CC#CC#CCCCC#CC#C[C@H](COS(O)(=O)=O)OS(O)(=O)=O | 492.1 |
| ZINC06924906 | COc1ccc(cc1)C1C[C@@H](c2cccc(c2)N(=O)=O)N(c2nc(c3ccccc3)c3ccccc3n2)N=1 | 501.2 |
| ZINC08444934 | CC(C)OC(c1c2CCCCc2sc1NC(c1cc2N[C@H](C[C@@H](C(F)(F)F)n2n1)c1ccc(cc1)[Br])=O)=O | 610.1 |
| ZINC08566718 | CC(C)C(Nc1c2cc(c3cccc(c3F)F)ncc2n[nH]1)=O | 316.1 |
| ZINC08687745 | C[C@H]1CCCN(C1)c1ccc(cc1N(=O)=O)C(=O)OCC(N1c2ccccc2Sc2ccccc12)=O | 503.2 |
| ZINC08763339 | CCOc1cc(C=C2C(N(CCCCCC(O)=O)C(=S)S2)=O)ccc1OCc1ccc(cc1)[Cl] | 519.1 |
| ZINC08845759 | Cc1ccc(cc1)[C@H]1c2c(c3ccc(C)cc3O)n[nH]c2C(N1CC=C)=O | 359.2 |
| ZINC08857198 | COc1cc(cc(c1OC)OC)c1c(C#N)c(C=Cc2ccc(cc2)F)nc2c3ccccc3C(c12)=O | 492.1 |
| ZINC09085826 | C(CCCCCN=C1C(c2ccccc2C([C@@H]1n1c2ccccc2nn1)=O)=O)CCCCC(O)=O | 474.2 |
| ZINC09216265 | [H][C@]12CC(c3ccc(cc3)OCC=C)=NN2[C@H](c2ccc(cc2)OCC)Oc2ccc(cc12)[Br] | 504.1 |
| ZINC09302584 | CCOP(c1ccccc1OCCOc1ccccc1P(O)(=O)OCC)(O)=O | 430.1 |
| ZINC09478505 | C1CCCN(CC1)S(c1cccc(c1)NC(Cc1c(cccc1[Cl])F)=O)(=O)=O | 424.1 |
| ZINC09668909 | Cc1ccc(CN2C(c3ccccc3[C@@H]2c2c3ccccc3n(C)c2c2cc(ccc2OC)OC)=O)o1 | 492.2 |
| ZINC10156626 | Cc1c2C(CCCc2nc(Nc2ccc(cc2)OC)n1)=O | 283.1 |
| ZINC10459406 | CC(C)N(C(C)C)C(Cn1cc(c2ccccc12)SCC(NCCc1ccc(c(c1)OC)OC)=O)=O | 511.3 |
| ZINC11635350 | Cc1cc2c(cc1S(N1CCOCC1)(=O)=O)NC(N2)=O | 297.1 |
| ZINC12300587 | CN(Cc1nc2cc(ccc2[nH]1)[Cl])C(CCc1nnc(C2CCCCC2)o1)=O | 401.2 |
| ZINC12402126 | CC1(C)CC(=C(C(CCCN2C(c3ccccc3C2=O)=O)=NCCc2ccccc2)C(C1)=O)O | 458.2 |
| ZINC12522595 | Cc1cccc(C(Nc2ccccc2N2CCOCC2)=O)n1 | 297.1 |
| ZINC12649152 | CCCC[C@@H](C(O)=O)SC1NC(C(Cc2cccc3ccccc23)=C(c2ccccc2)N=1)=O | 458.2 |
| ZINC12960152 | C(CN1C=C(C=NOCc2cccc(c2)[Cl])C(=C(Cc2ccccc2)C1=O)O)c1c[nH]c2ccccc12 | 511.2 |
| ZINC13016585 | CN(OC)S(c1ccc(c(c1)C(=O)OCc1c2ccccc2c2ccccc2n1)[Br])(=O)=O | 514.0 |
| ZINC13424914 | Cc1ccc(cc1C)N[C@@H]1[C@H]([C@@H]([C@@H]([C@@H](CO)O1)O[C@H]1[C@H]([C@@H]([C@@H]([C@H](CO)O1)O)O)O)O)O | 445.2 |
| ZINC13442292 | COc1ccccc1c1ccc(C[C@@H](C(O)=O)NC([C@]2(CCCO2)c2cccc(c2)C(F)(F)F)=O)cc1 | 513.2 |
| ZINC13458809 | CCc1ccc(cc1)[C@@H]1CC(=C(C=NCCN2CCN(CC2)C(NCC=C)=S)C(C1)=O)O | 454.2 |
| ZINC13473404 | CC(C)c1ccc(cc1)[C@H]1C(=C(c2ccc(cc2)OC)c2cc(ccc2O1)OC(C)C)C(O)=O | 458.2 |
| ZINC13488123 | CCCC[C@@H](C(=O)OC)N1C(=CN(CC(O)=O)C1=O)c1cccc(c1)Oc1ccc(cc1)C(C)(C)C | 494.2 |
| ZINC13521203 | [H][C@]12CC[C@]([H])(C1)[C@H]([C@@H]2CC=CCCCC(O)=O)NS(c1ccc(c(c1)N(=O)=O)OC)(=O)=O | 452.2 |
| ZINC13588144 | COc1cc(ccc1Cc1cn(C(c2ccccc2)c2ccccc2)c2ccc(cc12)S(C)(=O)=O)C(O)=O | 525.2 |
| ZINC13742379 | CC(C)=CCCC(C)=CCCC(C)(C)CCNC([C@H](CP(O)(O)=O)C(O)=O)=O | 417.2 |
| ZINC13756106 | CCc1nc2c(C)cc(C)nc2n1Cc1ccc(cc1)[C@@H](CC(O)=O)c1ccccc1 | 413.2 |
| ZINC13781080 | C(CCC[C@H](C[C@](CC(O)=O)(C(O)=O)O)O)CCc1ccc(cc1n1cccc1)[Cl] | 437.2 |
| ZINC13801841 | c1ccc(cc1)C([C@@H](c1ccccc1)OC(c1ccc2c(c1)nc1c3ccccc3c3ccccc3c1n2)=O)=O | 518.2 |
| ZINC13832866 | CCCN(Cc1ccc(cc1)c1ccccc1c1nn[nH]n1)c1c(cccn1)NC(C(=O)OCC)=O | 485.2 |
| ZINC14184176 | Cc1cc2ncc(C(Nc3cc(cc(c3)C(F)(F)F)C(F)(F)F)=O)c(C)n2n1 | 402.1 |
| ZINC14632500 | CC(C)(c1ccccc1)c1ccc(cc1)O[C@H]1C[C@@H](C(O)=O)N(C1)C(=O)OC(C)(C)C | 425.2 |
| ZINC14861931 | C1CC1Nc1ccc(cc1N(=O)=O)C(Nc1ccc(cc1)c1nc2ccccc2[nH]1)=O | 413.1 |
| ZINC14943303 | C(C[C@@H]1[C@H]([C@@H](Cc2ccccc2)N(Cc2cccc(c2)C(O)=O)C(N1Cc1cccc(c1)C(O)=O)=O)O)c1ccccc1 | 578.2 |
| ZINC15068482 | Cc1ccccc1CN1CCc2c(C(=O)OC)c(sc2C1)S(Nc1ccc(cc1)F)(=O)=O | 474.1 |
| ZINC15962202 | [H][C@@]12C[C@@](C)(NC(C1=C(C=Cc1ccc(c(c1OC)OC)OC)O)=O)Oc1ccccc12 | 423.2 |
| ZINC17303810 | CC(C)Oc1c(cc(cc1[Cl])C(Nc1nc2ccccc2[nH]1)=O)OC | 359.1 |
| ZINC18475154 | CCN(CC)S(c1ccc(c(c1)N(=O)=O)OCC(NCCc1ccc(cc1)C(C)C)=O)(=O)=O | 477.2 |
| ZINC19893988 | CC(C)Cc1ccc(cc1)c1cc(cc(c2ccc3c(c2)N(CCC(O)=O)C(CO3)=O)n1)c1ccc(C)cc1 | 520.2 |
| ZINC20421483 | CCOC(=C(C(C(F)(F)F)=O)[C@H](C)C(=C(C(F)(F)F)O)C(=O)OCC)O | 394.1 |
| ZINC20436566 | C1[C@@H]2C[C@@H]3C[C@H]1CC(C2)(C3)NC(Nc1ccc(cc1)C(C(F)(F)F)(C(F)(F)F)O)=O | 436.2 |
| ZINC20664765 | C(c1cccc(c1)F)S(C[C@@H](C(O)=O)Nc1c(cc(cc1N(=O)=O)C(O)=O)N(=O)=O)(=O)=O | 471.0 |
| ZINC20784163 | CC(C)n1c2c(cn1)[nH]c(c1ccc(c(c1)[Cl])OC)n2 | 290.1 |
| ZINC20942698 | CCOc1ccc(CCNCc2cccn2c2nnc(N3CCC(CC3)C(O)=O)s2)cc1OCC | 499.2 |
| ZINC22006540 | CC(C)CCC(C[C@@H](c1ccccc1C(O)=O)Nc1ccc2ccc(nc2n1)[Cl])=O | 425.2 |
| ZINC22605663 | Cc1ccc(cc1S(N1CCN(CC1)Cc1nc(cs1)c1ccc(cc1)[Cl])(=O)=O)N(=O)=O | 492.1 |
| ZINC22994119 | C1COCCC1C(Nc1cc(cc2cccnc12)[Cl])=O | 290.1 |
| ZINC23138061 | C(CNC(Cc1cc(nc(n1)SCc1ccc(cc1)F)O)=O)c1ccc(cc1)F | 415.1 |
| ZINC24615371 | CCCCN1CCS(c2c1c(nc(c2[Cl])Oc1ccccc1F)Oc1ccc(cc1)F)(=O)=O | 494.1 |
| ZINC25183531 | c1c(C(O)=O)c(C(C(F)F)(F)F)n(c2nc(cs2)C(O)=O)n1 | 339.0 |
| ZINC26015061 | CCCC(CC[C@H]1CCCc2c1cccc2OCC(O)=O)=NOC(c1ccccc1)c1ccccc1 | 485.3 |
| ZINC26186090 | CC(C)C[C@@H](C(N1CCC[C@H]1C(O)=O)=O)OP(CCCCc1ccccc1)(O)=O | 425.2 |
| ZINC26378892 | Cc1cc(C)cc(c1)C(N(C)[C@H](Cc1ccc(cc1)c1ccno1)C(N[C@@H](Cc1c[nH]c2ccccc12)C(=NS(C=C)(=O)=O)O)=O)=O | 653.2 |
| ZINC26388276 | CC(C)[C@]1(CCc2ccsc2CO)CC(C(=C(O)O1)Sc1cc(C)c(CO)cc1C(C)(C)C)=O | 504.2 |
| ZINC26402173 | CCCCc1nc(c(C(O)=O)n1Cc1ccc(cc1)c1ccccc1S(N=C(c1ccccc1)O)(=O)=O)SC | 563.2 |
| ZINC26567020 | CC(C)(C)c1cc(cc(c1O)C(C)(C)C)SC(C)(C)Sc1cc(c(c(c1)C(C)(C)C)OC(C(O)=O)=O)C(C)(C)C | 588.3 |
| ZINC26580061 | C(CC(O)=O)C[C@@H](C(O)=O)NC(=O)Oc1cc(c(c(c1)F)N(CC[Cl])CC[Cl])F | 456.1 |
| ZINC26666500 | CCCCc1ccc(COc2ccc3c4c2C[C@H](C)Sc4c(CC(C)(C)C(O)=O)n3Cc2ccc(cc2)[Cl])nc1 | 576.2 |
| ZINC26669662 | Cc1c(c2ccc(cc2)F)c(C=CP(C[C@@H](CC(O)=O)O)(O)=O)c(C2CC2)nc1c1ccccc1 | 495.2 |
| ZINC26818682 | COc1ccc(CCOC(c2ccc(cc2)[Cl])(c2ccc(cc2)[Cl])[C@H](C(O)=O)Oc2nc3CCCc3c(n2)OC)cc1OC | 638.2 |
| ZINC26992612 | CC(C)(C)c1ccc(cc1)S(Nc1c(c2ccc(CO)cc2)c(ncn1)OCCOc1ncc(cn1)[Br])(=O)=O | 613.1 |
| ZINC27523440 | [H][C@]12CC[C@]3(C)[C@]([H])(CC[C@]4([H])[C@@]5([H])[C@@H](CC[C@@]5(CC[C@@]34C)COC(CCC(O)=O)=O)C(C)=C)[C@@]2(C)CC[C@@H](C1(C)C)OC(CCC(O)=O)=O | 642.4 |
| ZINC27527290 | CCCc1ccc(C[C@@](C)(C(=NS(C(F)(F)F)(=O)=O)O)Oc2ccc(cc2)C(C)C)cc1 | 471.2 |
| ZINC27561937 | CCOc1cc(CCNC(c2cc(ccc2N2CCCCCCCC2)[Cl])=O)ccc1C(O)=O | 472.2 |
| ZINC27638993 | C(c1ccc(cc1)O)[C@@H](C(O)=O)N(Cc1ccccc1c1ccccc1)C(C=Cc1ccc2c(c1)OCO2)=O | 521.2 |
| ZINC27711148 | CC(C)c1ccccc1CN1C(C(=C(c2ccc(cc2)OC)c2c1c1ccccc1o2)C(O)=O)=O | 467.2 |
| ZINC28116754 | CCCCC1=NC2(CCCC2)C(N1Cc1ccc(c(COCC)c1)c1ccccc1S(Nc1c(C)c(C)no1)(=O)=O)=O | 592.3 |
| ZINC28130250 | C[C@@]1(Cc2cc(c(c(c2C1=O)[Cl])[Cl])OCCOc1ccc(cc1)c1nn[nH]n1)C1CCCC1 | 486.1 |
| ZINC28462262 | CC(C)(C)Cn1ccc2c(c(ccc12)OCCCCOc1ccc(cc1)c1nn[nH]n1)[Br] | 497.1 |
| ZINC28824000 | CC(C)[C@@]1(C)C(N=C(NC23C[C@@H]4C[C@@H](C[C@@H](C4)C3)C2)O1)=O | 290.2 |
| ZINC28891599 | CCc1ccc(cc1)c1ncc(c(n1)N(C)CCCOc1ccc2c(CC[C@H]2CC(O)=O)c1)F | 463.2 |
| ZINC28972042 | CCCCCN(C(Nc1ccc(cc1)C(C)C)=O)[C@@H]1Cc2ccc(cc2C1)SC(C)(C)C(O)=O | 482.3 |
| ZINC29061350 | CCOC(c1c(C(=O)OCC)nn2c1c1cc(c(cc1nc2O)[Cl])NC(N[C@@H](Cc1ccccc1)C(=O)OC)=O)=O | 583.1 |
| ZINC29125443 | CCOc1c2C(N(Cc2c(c2c1cccn2)OCC)c1ccc(CS(N=C(Cc2ccccc2F)O)(=O)=O)cc1C)=O | 591.2 |
| ZINC29128842 | [H][C@@]12CC=C([C@@H](C)SCC(CC)(CC)O)[C@@]2(C)CCCC1=CC=C1C[C@H](C[C@@H](C1)O)O | 434.3 |
| ZINC29279911 | COc1ccc2c(c1)[nH]c(n2)S[C@H]1CCCCCC1=O | 290.1 |
| ZINC29480471 | CCCN1C([C@H](NC(Nc2ccc(cc2)N2CCC(CC2)N2CCCCC2)=O)N=C(C2CCCCC2)c2ccccc12)=O | 584.4 |
| ZINC31703631 | CN(C)c1c(cccn1)C(N[C@H]1CCOc2ccccc12)=O | 297.1 |
| ZINC31805023 | c1ccc(cc1)Nc1ccc(cc1N(=O)=O)C(Nc1nnc2ccccn12)=O | 374.1 |
| ZINC32962265 | CCS(Nc1ccc(c(c1)C(O)=O)[N@]1CC[C@H](CCN2CCOCC2)CC1)(=O)=O | 425.2 |
| ZINC34802411 | CCOC(=C1C(CS(c2ccc(cc2)[Cl])(=O)=O)=NC(C)=C(C#N)[C@@H]1c1ccccc1C(F)(F)F)O | 524.1 |
| ZINC35684025 | CCC(N1[C@H](CC(c2ccccc2NS(c2ccccc2)(=O)=O)=N1)c1ccc(cc1)F)=O | 451.1 |
| ZINC36124819 | [H][C@]12CCO[C@]2([H])OC[C@@H]1OC(N[C@@H](Cc1ccccc1)[C@H](CN1CCCCCCCCCCCC[C@H](c2ccccc2)OC1=O)O)=O | 636.4 |
| ZINC36184595 | [H][C@]12CC[C@]3([H])[C@]4([H])CC[C@@H]([C@H](C)CCC(NCC(O)=O)=O)[C@@]4(C)CC[C@@]3([H])[C@@]2(C)Cc2c[nH]nc2C1 | 455.3 |
| ZINC36268381 | CC[C@H](CS[C@H](CCc1ccccc1C#N)c1cccc(CCc2ccc3ccc(cc3n2)[Cl])c1)C(O)=O | 542.2 |
| ZINC36351473 | CCCCC1(CCCC)CS(c2ccc(cc2[C@@H](c2cccc(CS(O)(=O)=O)c2)[C@H]1O)N(C)C)(=O)=O | 537.2 |
| ZINC36646873 | C1Cc2ccc(cc2N(C1)S(c1ccc(cc1)F)(=O)=O)NS(c1ccccc1N(=O)=O)(=O)=O | 491.1 |
| ZINC36779174 | CC1(C)C(=CC(COC(c2ccccc2NS(C)(=O)=O)=O)=O)N(C)c2ccccc12 | 428.1 |
| ZINC38741283 | CC(C)(C)c1cc(NC(c2ccc(cc2[Cl])F)=O)n(c2nc3CCCCc3c(n2)O)n1 | 443.2 |
| ZINC40628870 | C[C@](CNS(c1ccc(c(c1)[Cl])OC)(=O)=O)(c1ccc(cc1)F)O | 373.1 |
| ZINC40764151 | CC(C)=CCCC(C)=CCCC(C)=CCSC[C@@H](C(O)=O)NC(C12C[C@H]3C[C@H](C[C@H](C3)C2)C1)=O | 487.3 |
| ZINC40880885 | Cc1ccc(cc1)c1nc(c2ccc(c(c2)F)N2CCC[C@H](C2)C(N2CCN(CC2)C2CCCCC2)=O)no1 | 531.3 |
| ZINC41530225 | Cc1ccc2c(c1)c(c(C#N)nn2)Nc1cccc(c1)OC | 290.1 |
| ZINC42190928 | CC(C)N(CCN1CCOCC1)S(c1ccc(cc1)Nc1nc2c(C)cc(cc2nn1)c1cc(ccc1[Cl])O)(=O)=O | 596.2 |
| ZINC43904937 | C1CC2(CCC1=NNc1ccc(cc1)N(=O)=O)OCCO2 | 291.1 |
| ZINC43920140 | CCCCCCCCc1c(C=C[C@H](C[C@H](CC(O)=O)O)O)c(C(C)C)sc1c1ccccc1 | 472.3 |
| ZINC43929954 | CO[C@H]1CCCCC1=NNc1c(c(nc(c1F)F)F)F | 291.1 |
| ZINC47366883 | Cc1ccc(cc1)NC(CS[C@H](C)C(=C(C#N)C(c1ccccc1F)=O)O)=O | 398.1 |
| ZINC48412257 | CC(C)(C)C(CC1NC(c2c3CCCc3sc2N=1)=O)=O | 290.1 |
| ZINC48537647 | CC(C)c1cc(C(=NS(Cc2ccc(cc2)N(=O)=O)(=O)=O)O)nn1C(C)(C)C | 408.1 |
| ZINC48820056 | C(C(C(C#N)=C(Nc1ccc(cc1)F)O)=O)N1C=C(C=C(C1=O)[Cl])C(F)(F)F | 415.0 |
| ZINC48878276 | Cc1c(cc(c2nc3cc(c(cc3[nH]2)F)F)o1)C(=O)OC | 292.1 |
| ZINC49004577 | Cc1ccc2c(c(cnc2n1)C(N1CCCC1)=O)Nc1ccc(cc1)Oc1ccccc1 | 424.2 |
| ZINC49068987 | CC(CCC[C@H](C)C=C1C(=C(C)C(=O)O1)O)=CCC[C@](C)(CCCC1CN(CC(O)=O)C(C=1)=O)O | 489.3 |
| ZINC49071684 | C(C[C@](CO)(COP(O)(O)=O)N)c1ccc(cc1)c1ccc(cc1F)SCc1ccccc1 | 491.1 |
| ZINC49072205 | CSCC[C@@H](C(O)=O)NC(c1cc2c3ccccc3n(CCCc3ccccc3)c2cn1)=O | 461.2 |
| ZINC49162716 | CCC[C@@H](C(N(CCCCCC(O)=O)c1cc(C(O)=O)[nH]n1)=O)c1ccccc1 | 401.2 |
| ZINC49679552 | COc1cc2CC[C@@H](C3=CC(C(=CC=C3c2c(c1OC)OC)SC)=O)NC(CCCSSCCCC(O)=O)=O | 593.2 |
| ZINC49694839 | C[C@H]([C@H]1CN(CCN1Cc1ccccc1)C(Nc1cccc2ccccc12)=O)Nc1nccc(n1)N1C=N[C+]2=CC=CC=[C+]1=2 | 582.3 |
| ZINC49723260 | Cc1ccc(cc1S(NC[C@H]1CCCO1)(=O)=O)c1c2ccccc2c(Nc2ccc(cc2)C(N2CCCCC2)=O)nn1 | 585.2 |
| ZINC51740554 | C(C(C(C#N)=C(Nc1ccccc1[Br])O)=O)SCc1cccs1 | 408.0 |
| ZINC52617929 | CCCC[C@H]1CC=C(CNC(CCC)=O)C(N1Cc1ccc(cc1)c1ccccc1c1nnn[nH]1)=O | 486.3 |
| ZINC52835559 | C1C(NCCN(C[C@@H](c2ccc3ccccc3c2)O)[C@@H]1c1ccccc1)=O | 360.2 |

***Supplementary table*** ***S5. Experimentally tested VLS hit compounds. Experimentally confirmed hits for CysLT1R are in bold, for CysLT2R are in italic.***

| **BRI-ID** | **SMILES** | **MW** | **cLogP** | **cLogS** | **Receptor model** | **Enamine ID** | **CysLT1R, % IP1 production at 30 mM,** | | **CysLT2R, % IP1 production at 30 mM,** | |
| --- | --- | --- | --- | --- | --- | --- | --- | --- | --- | --- |
|  |  |  |  |  |  |  | **Mean** | **±SEM** | **Mean** | **±SEM** |
| BRI-12301 | C(CCC(Nc1ccc(cc1)C(N)=O)=O)CC1C2C(CS1)NC(N2)=O | 362.1 | 0.7 | -1.8 | CysLT2R crystal structure | Z223846660 | 91.51 | 14.16 | 88.13 | 33.73 |
| BRI-12302 | C1CCN(C(C1)CCC(O)=O)C(C1CCc2c(C1)cncn2)=O | 317.2 | 0.4 | -1.0 | CysLT1R LiBERO-optimized model | Z2446491828 | 87.37 | 7.56 | 70.03 | 22.37 |
| BRI-12303 | Cn1cc(cc1C(N)=O)S(N[C@@H]1CC[C@@H](C1)C(O)=O)(=O)=O | 315.1 | -0.4 | -2.0 | CysLT1R LiBERO-optimized model | Z2446077013 | 77.88 | 11.17 | 86.48 | 14.19 |
| BRI-12304 | C1CN(Cc2ccccc12)S(Nc1cccc(c1C(O)=O)F)(=O)=O | 350.1 | 3.3 | -3.6 | CysLT1R LiBERO-optimized model | Z1185227208 | 83.80 | 6.98 | 106.20 | 8.16 |
| BRI-12305 | C(C(c1ccc(cc1)F)NC(Cn1c2ccccc2cn1)=O)C(O)=O | 341.1 | 2.2 | -2.6 | CysLT1R LiBERO-optimized model | Z1443671314 | 93.10 | 3.20 | 108.90 | 10.87 |
| BRI-12306 | C[C@@H]1C[C@H](C(Nc2cccc(c2)NC(c2cccs2)=O)=O)O[C@H]1C(O)=O | 374.1 | 2.0 | -2.6 | CysLT2R LiBERO-optimized model | Z2755267083 | 81.25 | 6.15 | 112.50 | 8.38 |
| BRI-12307 | C1CN2C(CN1C(c1ccccc1NC(c1cccs1)=O)=O)C(NC2=O)=O | 384.1 | 1.2 | -2.3 | CysLT2R crystal structure | Z1345982171 | 84.62 | 6.73 | 109.90 | 10.30 |
| BRI-12308 | CC1CC(CN(C1)C(Nc1cccc(c1)NC(c1ccccc1)=O)=O)C(O)=O | 381.2 | 2.6 | -3.1 | CysLT2R LiBERO-optimized model | Z2376767126 | 95.61 | 17.01 | 101.40 | 12.98 |
| BRI-12309 | C1C[C@H](C[C@H]1C(Nc1cccc(c1)c1nc2ccccc2[nH]1)=O)C(O)=O | 349.1 | 3.2 | -3.6 | CysLT2R LiBERO-optimized model | Z2352609311 | 87.85 | 10.70 | 87.42 | 11.75 |
| BRI-12310 | C1CCN(C(C1)CCC(O)=O)C(Cc1c[nH]c2cc(ccc12)F)=O | 332.2 | 2.0 | -2.4 | CysLT1R LiBERO-optimized model | Z1609741268 | 77.29 | 6.84 | 91.59 | 11.70 |
| BRI-12311 | C(C(c1ccccc1)NC(c1ccc(c(c1)N1CCNC1=O)[Cl])=O)C(O)=O | 387.1 | 2.2 | -3.3 | CysLT1R LiBERO-optimized model | Z1443609979 | 77.51 | 10.39 | 90.99 | 11.60 |
| BRI-12312 | C1CCN(C(C1)c1cn[nH]c1)C(Nc1ccc2COCc2c1)=O | 312.2 | 1.9 | -2.5 | CysLT1R LiBERO-optimized model | Z1671782546 | 77.41 | 17.64 | 87.18 | 25.10 |
| BRI-12313 | C(C(NCC(O)=O)=O)c1ccc(cc1)NC(c1c2ccccc2[nH]n1)=O | 352.1 | 0.9 | -2.0 | CysLT2R crystal structure | Z1603470413 | 88.59 | 6.83 | 110.30 | 10.91 |
| BRI-12314 | C(Cc1nc2c3ccccc3nc(n2n1)SCC(O)=O)c1ccccc1 | 364.1 | 3.4 | -3.5 | CysLT1R crystal structure | Z235352447 | 66.65 | 14.74 | 96.87 | 6.75 |
| BRI-12315 | C(c1cccnc1)N1C(c2ccc(cc2)O)c2c(c3ccccc3O)n[nH]c2C1=O | 398.1 | 3.1 | -3.2 | CysLT1R LiBERO-optimized model | Z1213671665 | 83.34 | 7.66 | 104.90 | 9.99 |
| BRI-12316 | COc1ccc(C2CC(c3cccs3)N(C(CCC(O)=O)=O)N=2)c(c1)O | 374.1 | 2.6 | -3.0 | CysLT2R LiBERO-optimized model | Z2218300755 | 91.00 | 12.38 | 90.03 | 15.26 |
| BRI-12317 | C(C(O)=O)NC(c1ccc(cc1)NC(c1cc2ccccn2c1)=O)=O | 337.1 | 1.6 | -2.0 | CysLT1R crystal structure | Z1444864410 | 86.38 | 12.21 | 89.25 | 11.21 |
| BRI-12318 | CC(Nc1ccccc1OS(c1cccc2cc(C)cnc12)(=O)=O)=O | 356.1 | 2.3 | -3.0 | CysLT1R crystal structure | Z271163560 | 74.12 | 14.13 | 88.18 | 14.77 |
| BRI-12319 | CC(c1ccc(cc1)OCC(NC(CC(O)=O)c1cccs1)=O)=O | 347.1 | 1.3 | -1.8 | CysLT2R LiBERO-optimized model | Z364313594 | 76.44 | 15.20 | 78.43 | 18.12 |
| BRI-12320 | C1CC(CN(CCC(O)=O)C(c2cc(c3ccccc3[Cl])[nH]n2)=O)OC1 | 377.1 | 1.5 | -2.8 | CysLT1R LiBERO-optimized model | Z1444599077 | 81.44 | 8.32 | 90.55 | 16.87 |
| BRI-12321 | C1CCC(C1)NC(c1ccc(cc1)NC(C1C2CC(C=C2)C1C(O)=O)=O)=O | 368.2 | 2.5 | -2.9 | CysLT2R LiBERO-optimized model | Z735908538 | 87.85 | 3.74 | 95.09 | 14.05 |
| BRI-12322 | CNC(c1cccc(c1)NC(CN1c2cccc3cccc(c23)S1(=O)=O)=O)=O | 395.1 | 2.5 | -3.3 | CysLT1R LiBERO-optimized model | Z29130914 | 69.84 | 11.33 | 79.08 | 23.21 |
| BRI-12323 | C(C(O)=O)NC(c1ccc(cc1)NS(c1cccs1)(=O)=O)=O | 340.0 | 0.7 | -1.9 | CysLT1R crystal structure | Z45581680 | 80.52 | 5.66 | 100.00 | 12.15 |
| BRI-12324 | C1CN(CC=C1c1ccccc1)S(c1ccc(c(c1)C(O)=O)O)(=O)=O | 359.1 | 4.3 | -3.9 | CysLT2R crystal structure | Z45578234 | 82.12 | 11.71 | 67.44 | 21.20 |
| BRI-12325 | CC1=C(C(c2ccncc2)n2c(N1)nc(CCCO)n2)C(Nc1ccccc1)=O | 390.2 | 1.4 | -1.7 | CysLT1R crystal structure | Z1203159348 | 76.09 | 12.78 | 79.33 | 18.84 |
| BRI-12326 | CS(Cc1cccc(c1)NC(CC1(Cc2ccccc2C1)C(O)=O)=O)=O | 371.1 | 1.9 | -2.5 | CysLT2R LiBERO-optimized model | Z1213004308 | 79.32 | 14.36 | 70.98 | 17.48 |
| BRI-12327 | Cn1cc(C(N)=O)c(NC(CCc2cccc(c2)C(F)(F)F)=O)n1 | 340.1 | 1.3 | -2.5 | CysLT1R crystal structure | Z1580135885 | 68.50 | 6.22 | 73.39 | 16.96 |
| ***BRI-12328*** | ***CC1(C(NC(N1)=O)=O)c1ccc(cc1)C(Nc1nc(cs1)c1ccccc1)=O*** | ***392.1*** | ***3.5*** | ***-3.8*** | ***CysLT2R crystal structure*** | ***Z1334880971*** | ***21.01*** | ***14.40*** | ***38.10*** | ***17.25*** |
| BRI-12329 | C1C(C(Nc2ccc(cc2)C(NCC(O)=O)=O)=O)Oc2ccc(cc12)F | 358.1 | 1.2 | -2.1 | CysLT1R crystal structure | Z1444862339 | 88.83 | 4.52 | 87.75 | 14.96 |
| BRI-12330 | C(C(c1ccc(c(c1)F)[Cl])NC(c1cccc(c1)OCC(N)=O)=O)C(O)=O | 394.1 | 1.4 | -2.5 | CysLT2R LiBERO-optimized model | Z1603681406 | 72.07 | 6.96 | 93.36 | 14.76 |
| BRI-12331 | CN(CC(Nc1ccc2c(c1)cn[nH]2)=O)C1c2ccccc2S(N=1)(=O)=O | 369.1 | 1.7 | -2.7 | CysLT1R LiBERO-optimized model | Z261773904 | 84.33 | 4.07 | 92.77 | 14.48 |
| BRI-12332 | C1CN(CC=C1c1c[nH]c2ccccc12)C(c1ccc2nnnn2c1)=O | 344.1 | 2.1 | -2.8 | CysLT1R LiBERO-optimized model | Z1409330612 | 96.66 | 8.49 | 91.15 | 23.98 |
| BRI-12333 | Cc1cccc(c1)n1c(C)c(C(Nc2ccc3c(c2)NC(CO3)=O)=O)nn1 | 363.1 | 2.4 | -3.0 | CysLT1R crystal structure | Z729190622 | 77.51 | 16.90 | 70.52 | 13.95 |
| ***BRI-12334*** | ***Cc1cc(c(cc1S(Nc1ccc(c(c1)F)N1CCCC1)(=O)=O)C(O)=O)O*** | ***394.1*** | ***3.8*** | ***-3.7*** | ***CysLT2R crystal structure*** | ***Z751235032*** | ***47.28*** | ***13.82*** | ***57.16*** | ***13.49*** |
| BRI-12335 | C1Cc2c3C(N(CC(c4ccc5c(c4)NC(CO5)=O)=O)C=Nc3sc2C1)=O | 381.1 | 1.9 | -2.7 | CysLT1R crystal structure | Z154257036 | 73.75 | 10.67 | 74.82 | 14.90 |
| BRI-12336 | CC1CC1C(NCCC(Nc1cccc(c1n1cncn1)[Cl])=O)=O | 347.1 | 1.0 | -2.3 | CysLT2R LiBERO-optimized model | Z393059954 | 77.33 | 6.13 | 92.09 | 8.88 |
| BRI-12337 | Cn1c2ccc(cc2nc1CN1C=C(C=CC1=O)c1nnco1)[Cl] | 341.1 | 1.2 | -2.3 | CysLT1R crystal structure | Z666170944 | 71.95 | 10.89 | 91.32 | 14.43 |
| BRI-12338 | C1Cc2cc(ccc2NC1=O)NC(C(NCCc1ccc2c(c1)OCO2)=O)=O | 381.1 | 1.2 | -2.1 | CysLT2R crystal structure | Z437613398 | 66.42 | 10.10 | 95.51 | 12.44 |
| BRI-12339 | CC(C)c1c(cnn1c1ccccn1)C(Nc1ccc2c(c1)NC(N2)=O)=O | 362.1 | 3.6 | -3.9 | CysLT1R crystal structure | Z381297548 | 82.01 | 9.71 | 99.82 | 14.48 |
| BRI-12340 | C(C(O)=O)Oc1ccc(C=Cc2ccc3cccc(c3n2)O)cc1 | 321.1 | 3.6 | -3.6 | CysLT2R crystal structure | Z45957013 | 70.18 | 7.64 | 78.94 | 18.51 |
| BRI-12341 | C1CN(Cc2c3cc(ccc3[nH]c12)F)C(CN1C(NC(c2ccccc12)=O)=O)=O | 392.1 | 1.7 | -2.6 | CysLT1R LiBERO-optimized model | Z344107900 | 69.49 | 22.80 | 61.44 | 11.82 |
| BRI-12342 | CC(C(c1c[nH]c2ccccc12)=O)SC1=NNC(N1C1CC1)=O | 328.1 | 3.6 | -3.9 | CysLT1R LiBERO-optimized model | Z97434952 | 71.73 | 17.57 | 81.25 | 11.26 |
| BRI-12343 | CC(c1cc2c(cc1NC(COc1ccc(cc1C(N)=O)[Cl])=O)OCO2)=O | 390.1 | 2.1 | -3.3 | CysLT1R LiBERO-optimized model | Z97750052 | 73.73 | 19.66 | 88.87 | 15.61 |
| BRI-12344 | c1cc(cc(c1)NS(c1cc(cs1)C(O)=O)(=O)=O)NC(c1ccco1)=O | 392.0 | 2.7 | -3.2 | CysLT2R LiBERO-optimized model | Z237836840 | 72.53 | 9.31 | 96.85 | 15.07 |
| BRI-12345 | C(C1C(N=C(C2=Cc3ccccc3OC2=O)S1)=O)C(Nc1ccc(cc1)F)=O | 396.1 | 2.4 | -3.3 | CysLT1R LiBERO-optimized model | Z53088685 | 75.31 | 4.41 | 100.70 | 4.98 |
| BRI-12346 | C1C2C=CC1C(C2C(O)=O)C(Nc1cccc(c1)C(NCC(F)(F)F)=O)=O | 382.1 | 2.4 | -2.9 | CysLT2R LiBERO-optimized model | Z320945562 | 66.59 | 7.59 | 90.60 | 16.84 |
| BRI-12347 | CC(C(NC(N)=O)=O)Sc1nnc(c2c[nH]c3ccccc23)n1C1CC1 | 370.1 | 2.4 | -2.9 | CysLT2R crystal structure | Z92492347 | 79.74 | 2.57 | 103.80 | 10.35 |
| BRI-12348 | COc1ccc(cc1)C1CC(c2cccs2)N(C(CCC(O)=O)=O)N=1 | 358.1 | 2.4 | -2.6 | CysLT2R LiBERO-optimized model | Z295196818 | 89.51 | 9.55 | 106.70 | 12.21 |
| BRI-12349 | C(C(NC(N)=O)=O)OC(c1ccccc1Nc1cccc(c1)C(F)(F)F)=O | 381.1 | 3.7 | -4.2 | CysLT2R crystal structure | Z18864152 | 80.03 | 19.49 | 70.53 | 12.63 |
| BRI-12350 | Cc1cccc(c1)c1nnc(n1CC(O)=O)SCc1ccc(cc1)C(N)=O | 382.1 | 1.3 | -1.8 | CysLT2R crystal structure | Z166092398 | 68.38 | 11.78 | 79.84 | 11.39 |
| BRI-12351 | CC(C(N1CC(Nc2ccccc12)=O)=O)Sc1ccccc1C(O)=O | 356.1 | 2.6 | -2.9 | CysLT1R LiBERO-optimized model | Z242710376 | 77.56 | 12.16 | 82.64 | 14.96 |
| BRI-12352 | C(CNS(c1ccc(c(c1)C(O)=O)O)(=O)=O)c1ccc(cc1)[Cl] | 355.0 | 3.9 | -4.2 | CysLT2R LiBERO-optimized model | Z45690654 | 73.41 | 19.23 | 87.80 | 7.79 |
| BRI-12353 | C=C1c2ccccc2C(N1CCC(Nc1cc(cc(c1)C(N)=O)C(N)=O)=O)=O | 378.1 | 1.4 | -2.3 | CysLT1R LiBERO-optimized model | Z229587984 | 83.42 | 4.76 | 96.79 | 10.14 |
| BRI-12354 | C(C(Nc1cccc(C=Cc2ccccn2)c1)=O)N1C=CC(NC1=O)=O | 348.1 | 1.7 | -2.1 | CysLT2R crystal structure | Z231487084 | 72.68 | 10.96 | 92.82 | 8.90 |
| BRI-12355 | C(C(c1ccccc1)c1c[nH]c2ccccc12)Nc1ccc2nnnn2n1 | 355.2 | 3.5 | -3.6 | CysLT1R LiBERO-optimized model | Z220408858 | 76.84 | 12.17 | 91.85 | 10.77 |
| BRI-12356 | COc1ccc(cc1F)NC(N1C2CCC1c1cncnc1C2)=O | 328.1 | 1.8 | -2.3 | CysLT2R crystal structure | Z2798534718 | 92.94 | 7.62 | 104.60 | 12.40 |
| BRI-12357 | CN(CC(Nc1ccc(cc1)OC)=O)C(c1ccccc1n1cnnn1)=O | 366.1 | 1.6 | -2.3 | CysLT2R LiBERO-optimized model | Z26932290 | 77.00 | 14.71 | 65.66 | 11.49 |
| BRI-12358 | CC(C(N1CCNC1=O)=O)Sc1nc(c2ccccc2n1)Nc1ccccc1 | 393.1 | 3.4 | -3.7 | CysLT2R crystal structure | Z113220656 | 58.01 | 14.55 | 73.45 | 11.91 |
| ***BRI-12359*** | ***C(C(Nc1c(ccs1)C(N)=O)=O)Sc1nnc(c2c[nH]c3ccccc23)o1*** | ***399.0*** | ***1.5*** | ***-2.3*** | ***CysLT1R LiBERO-optimized model*** | ***Z19303751*** | ***5.59*** | ***17.13*** | ***36.23*** | ***10.03*** |
| BRI-12360 | Cc1ccc(cc1)c1c(c2ccc(C)cc2)nnc(n1)SCC(NC(N)=O)=O | 393.1 | 3.2 | -3.6 | CysLT1R crystal structure | Z16547154 | 74.27 | 8.51 | 90.47 | 14.26 |
| BRI-12361 | C(C(c1ccccc1)NC(c1ccc2c(cc[nH]2)c1)=O)C(O)=O | 308.1 | 2.5 | -2.9 | CysLT2R LiBERO-optimized model | Z1152457793 | 83.40 | 3.28 | 91.26 | 9.41 |
| *BRI-12401* | *C(c1ccccc1)N1C(NC(c2cc(cnc12)C(Nc1cc2cc[nH]c2nc1)=O)=O)=O* | *412.1* | *3.2* | *-3.5* | *CysLT2R LiBERO-optimized model* | *Z3718279219* | *89.55* | *14.70* | *35.63* | *18.16* |
| *BRI-12402* | *Cn1c(CC(Nc2ccc(cc2)N2CCNC2=O)=O)nc(C2CC2)n1* | *340.2* | *1.1* | *-2.1* | *CysLT2R crystal structure* | *Z2453271081* | *66.82* | *22.29* | *40.17* | *12.88* |
| BRI-12403 | c1ccc(c(c1)C(C(O)=O)NC(c1ccc(c(c1)NC(c1cccs1)=O)[Cl])=O)F | 432.0 | 3.3 | -3.3 | CysLT1R LiBERO-optimized model | Z1603667280 | 89.55 | 12.05 | 72.05 | 9.69 |
| BRI-12404 | CN(CCOc1ccc(cc1)OCc1ccccc1)C(C1=CC(Nc2ccc(cc12)F)=O)=O | 446.2 | 4.2 | -4.6 | CysLT2R LiBERO-optimized model | Z1239336123 | 83.82 | 8.50 | 67.28 | 20.73 |
| *BRI-12405* | *C1CC(c2ccccc2C1)OCC(Nc1cccc(c1)NC(NCc1ccncc1)=O)=O* | *430.2* | *3.5* | *-3.7* | *CysLT2R crystal structure* | *Z1972228976* | *70.01* | *17.42* | *40.63* | *16.99* |
| BRI-12406 | CC(C)c1cc(C(Nc2ccc(cc2)C(NCC(O)=O)=O)=O)nn1C | 344.1 | 0.9 | -2.1 | CysLT2R LiBERO-optimized model_final | Z1444867397 | 79.60 | 13.50 | 68.41 | 14.98 |
| BRI-12407 | CC(C)(C)CNC(c1ccc(cc1)C(Nc1ccccc1CC(O)=O)=O)=O | 368.2 | 3.6 | -3.5 | CysLT2R crystal structure | Z1444783145 | 82.99 | 19.94 | 70.40 | 10.90 |
| BRI-12408 | CCNC(CNC(c1cc(c[nH]1)c1csc(C)n1)=O)=O | 292.1 | 1.5 | -2.2 | CysLT1R crystal structure | Z117287020 | 101.80 | 16.39 | 62.29 | 17.46 |
| BRI-12409 | C(C(c1cccc(c1)OCc1ccccc1)O)NC(C1=CC(Nc2ccc(cc12)F)=O)=O | 432.1 | 3.0 | -3.6 | CysLT1R crystal structure | Z1423536727 | 73.29 | 15.58 | 62.33 | 22.52 |
| ***BRI-12410*** | ***C(c1ccccc1)N(Cc1nc(c2cc(cnc2)S(N)(=O)=O)no1)c1ccccc1*** | ***421.1*** | ***3.3*** | ***-3.4*** | ***CysLT1R LiBERO-optimized model*** | ***Z2234555377*** | ***45.23*** | ***18.67*** | ***46.73*** | ***8.46*** |
| **BRI-12411** | **Cc1cccc(COc2ccc(CNS(N(C)CCC(O)=O)(=O)=O)cc2)c1** | **392.1** | **2.8** | **-3.3** | **CysLT2R crystal structure** | **Z2437641363** | **55.92** | **8.88** | **57.31** | **13.36** |
| BRI-12412 | C(CC(O)=O)C(c1ccccc1)NC(c1cccc(c1)C(F)(F)F)=O | 351.1 | 3.1 | -3.6 | CysLT2R LiBERO-optimized model | Z1444480195 | 91.43 | 9.22 | 63.42 | 8.51 |
| *BRI-12413* | *C(C(O)=O)NC(c1ccc(cc1)S(Nc1ccc2c(c1)cns2)(=O)=O)=O* | *391.0* | *1.1* | *-2.5* | *CysLT1R crystal structure* | *Z1822566948* | *79.99* | *8.55* | *49.53* | *23.55* |
| BRI-12414 | C(C(Nc1cccc(c1)n1ccc(C(O)=O)n1)=O)c1cccc(c1)OCc1cccnc1 | 428.1 | 2.6 | -2.7 | CysLT2R crystal structure | Z1869834736 | 81.81 | 15.70 | 84.93 | 12.54 |
| BRI-12415 | C(CC(Nc1ccc(cc1)C(Nc1cccnc1)=O)=O)Cc1ccc2c(c1)NC(N2)=O | 415.2 | 2.7 | -3.0 | CysLT2R crystal structure | Z1144525004 | 85.08 | 10.42 | 56.80 | 14.42 |
| BRI-12416 | C1CN(CCC1c1cc2ccccc2[nH]1)C(c1cc2c(cc1[Cl])NC(CO2)=O)=O | 409.1 | 3.7 | -4.3 | CysLT1R crystal structure | Z3718279205 | 70.84 | 15.55 | 80.38 | 14.26 |
| ***BRI-12417*** | ***c1ccc(c(c1)C(N)=O)Oc1ccc(cc1)NC(c1ccc2nc(cn2c1)C(F)(F)F)=O*** | ***440.1*** | ***3.7*** | ***-4.3*** | ***CysLT1R LiBERO-optimized model*** | ***Z1715894643*** | ***54.73*** | ***19.39*** | ***74.42*** | ***11.60*** |
| BRI-12418 | C(c1ccc(C(N)=O)o1)NC(c1ccc(cc1O)NC(c1ccc(c(c1)[Cl])[Cl])=O)=O | 447.0 | 3.8 | -4.1 | CysLT1R crystal structure | Z1371601635 | 65.40 | 15.33 | 67.88 | 10.45 |
| BRI-12419 | C1Cn2c(CN1C(c1ccccc1NC(c1ccc(cc1)[Cl])=O)=O)ncn2 | 381.1 | 2.1 | -3.1 | CysLT2R LiBERO-optimized model | Z1721041912 | 85.67 | 8.63 | 48.10 | 18.92 |
| BRI-12420 | CC(C)NC(Nc1ccc(cc1)C(Nc1ccc(c(c1)[Cl])OCC(O)=O)=O)=O | 405.1 | 2.9 | -3.1 | CysLT2R LiBERO-optimized model | Z1603685831 | 91.65 | 7.71 | 64.19 | 19.42 |
| BRI-12421 | C(C(CNS(c1ccc(cc1)Oc1ccc(cc1)[Cl])(=O)=O)C(O)=O)C(O)=O | 413.0 | 1.7 | -2.7 | CysLT2R crystal structure | Z2178758040 | 82.61 | 11.06 | 57.26 | 14.59 |
| BRI-12422 | CC(C)OCC(Nc1ccc(cc1)c1nccc(C)n1)=O | 285.1 | 2.1 | -2.6 | CysLT2R crystal structure | Z1210269000 | 79.83 | 10.94 | 76.45 | 11.77 |
| BRI-12423 | CC(CN(C1CC1)C(Nc1ccc2C(CCCc2c1)=O)=O)O | 302.2 | 2.5 | -3.2 | CysLT1R crystal structure | Z2494912178 | 89.70 | 9.72 | 68.21 | 13.02 |
| ***BRI-12424*** | ***Cn1c2ccccc2nc1c1ccc(cc1)NC(c1cccc(c1)OCC(O)=O)=O*** | ***401.1*** | ***3.7*** | ***-3.9*** | ***CysLT2R crystal structure*** | ***Z1444195420*** | ***50.81*** | ***14.96*** | ***79.69*** | ***12.99*** |
| BRI-12425 | Cc1c(c(N)no1)S(Nc1ccc(cc1C(O)=O)N1CCCC1)(=O)=O | 366.1 | 1.2 | -2.6 | CysLT1R LiBERO-optimized model | Z2475281313 | 84.15 | 15.46 | 79.75 | 15.27 |
| *BRI-12426* | *C(CNC(c1cc(sc1)S(N)(=O)=O)=O)c1ccc(cc1)OCC(O)=O* | *384.0* | *0.4* | *-2.0* | *CysLT1R crystal structure* | *Z1445575601* | *72.63* | *13.05* | *41.76* | *8.37* |
| *BRI-12427* | *C(c1cccc(c1)OCC(O)=O)NC(c1cnc(c2ncccn2)s1)=O* | *370.1* | *0.7* | *-2.1* | *CysLT2R crystal structure* | *Z1445429628* | *78.65* | *8.27* | *42.37* | *3.16* |
| BRI-12428 | Cc1ccc(C(Nc2ccc(cc2)C(NCC(O)=O)=O)=O)c(c1)O | 328.1 | 1.6 | -2.5 | CysLT1R crystal structure | Z1539141440 | 90.35 | 10.38 | 59.53 | 10.54 |
| BRI-12429 | C1CC(C1)c1ccc(cc1)C(Nc1cccc(c1)S(NCC(O)=O)(=O)=O)=O | 388.1 | 2.1 | -2.9 | CysLT2R crystal structure | Z2655961345 | 72.30 | 11.00 | 68.04 | 14.45 |
| BRI-12430 | CN(C(c1ccc(cc1)C(Nc1ccsc1C(N)=O)=O)=O)c1ccccc1 | 379.1 | 1.9 | -2.5 | CysLT1R LiBERO-optimized model | Z1524772030 | 79.55 | 14.84 | 92.53 | 8.46 |
| BRI-12431 | CN(CCCc1cc(c2ccc(cc2)F)n[nH]1)S(c1c(cn[nH]1)C(O)=O)(=O)=O | 407.1 | 2.5 | -3.0 | CysLT2R LiBERO-optimized model | Z928038744 | 90.92 | 9.05 | 63.21 | 13.88 |
| BRI-12432 | COc1ccc(cc1)N(CCC(O)=O)S(c1csc(c2ccc(cc2)F)n1)(=O)=O | 436.1 | 2.9 | -3.4 | CysLT2R LiBERO-optimized model | Z1738953910 | 81.02 | 12.24 | 76.91 | 8.51 |
| BRI-12433 | C(C(O)=O)NC(c1ccc(cc1)NS(c1csc(c2ccc(cc2)[Cl])n1)(=O)=O)=O | 451.0 | 2.9 | -3.5 | CysLT1R crystal structure | Z1148083760 | 76.25 | 17.90 | 62.74 | 23.24 |
| BRI-12434 | CCN(CC)C(c1ccc(cc1)C(=O)OCc1cc(C(C)=O)no1)=O | 344.1 | 2.1 | -2.4 | CysLT2R crystal structure | Z2191458590 | 57.63 | 22.77 | 62.01 | 12.58 |
| **BRI-12435** | **Cc1ccccc1Nc1nc2c(cc(C(NCCc3nnc4C(NCCn34)=O)=O)s2)s1** | **453.1** | **2.3** | **-2.9** | **CysLT2R LiBERO-optimized model** | **Z3718279221** | **54.50** | **12.37** | **77.11** | **5.78** |
| BRI-12436 | C1COC(CN1C(Nc1cc(ccn1)[Br])=O)C(O)=O | 329.0 | 1.0 | -2.1 | CysLT1R crystal structure | Z2362306718 | 93.88 | 6.45 | 67.50 | 11.38 |
| BRI-12437 | Cc1ccc(cc1)C(c1ccccn1)NS(c1ccc(cc1)C(NCC(O)=O)=O)(=O)=O | 439.1 | 1.8 | -2.9 | CysLT1R crystal structure | Z1956958510 | 91.87 | 7.59 | 80.53 | 11.54 |
| BRI-12438 | Cc1cccn2cc(c3cccc(c3)NC(CN3C(NC(c4ccccc34)=O)=O)=O)nc12 | 425.1 | 3.9 | -4.1 | CysLT2R LiBERO-optimized model | Z3718279220 | 62.57 | 13.84 | 88.69 | 7.21 |
| BRI-12439 | C(C(C(O)=O)NS(c1csc(c2ccc(cc2)F)n1)(=O)=O)c1c[nH]c2c1cccn2 | 446.1 | 2.5 | -2.8 | CysLT2R crystal structure | Z1738105955 | 88.26 | 7.60 | 72.38 | 9.33 |
| BRI-12440 | C(C(O)=O)NC(c1ccc(cc1)NC(c1cc2ccccc2nc1)=O)=O | 349.1 | 1.3 | -2.2 | CysLT1R crystal structure | Z2910339104 | 95.21 | 12.78 | 75.03 | 11.74 |
| BRI-12441 | C(Cc1cccc(c1F)F)C(Nc1ccc(cc1)C(NCC(O)=O)=O)=O | 362.1 | 1.7 | -2.2 | CysLT1R crystal structure | Z1444864283 | 88.31 | 5.17 | 70.92 | 12.11 |
| BRI-12442 | C(Cn1cccc1)C(Nc1ccc(cc1)C(NCC(O)=O)=O)=O | 315.1 | 0.7 | -1.6 | CysLT1R crystal structure | Z1444865419 | 74.52 | 8.86 | 51.69 | 16.71 |
| BRI-12443 | COc1ccccc1OCc1ccc(C(N[C@@H](CC(O)=O)c2ccc(cc2)[Cl])=O)o1 | 429.1 | 3.9 | -4.5 | CysLT1R LiBERO-optimized model | Z1603675043 | 84.47 | 8.82 | 82.35 | 10.65 |
| BRI-12444 | Cc1cnnn1c1ccc(cc1)NC(c1cccc(c1)NC(CCCc1cccs1)=O)=O | 445.2 | 3.8 | -4.1 | CysLT1R crystal structure | Z3718279210 | 96.10 | 7.45 | 68.24 | 12.16 |
| BRI-12445 | CC(C)(C)n1nc(COC(C2c3ccccc3CCO2)=O)nn1 | 316.2 | 2.2 | -2.4 | CysLT2R crystal structure | Z2498641879 | 86.60 | 10.94 | 67.66 | 19.86 |
| BRI-12446 | C1CCC(CC1)NC(c1ccc(cc1O)NC(c1ccc(c(c1)S(NC1CC1)(=O)=O)F)=O)=O | 475.2 | 3.8 | -3.7 | CysLT2R crystal structure | Z3718279216 | 72.39 | 11.81 | 67.67 | 17.63 |
| BRI-12447 | C(c1c2ccccc2oc1C(O)=O)NC(c1ccc(cc1)NC(N)=O)=O | 353.1 | 2.0 | -2.5 | CysLT1R LiBERO-optimized model | Z1445235438 | 85.53 | 12.53 | 52.93 | 21.18 |
| BRI-12448 | Cc1c(CCC(N(C)c2ccc3cc[nH]c3c2)=O)c(C)n2c(c(cn2)C(N)=O)n1 | 390.2 | 1.4 | -2.5 | CysLT1R crystal structure | Z1944121471 | 95.90 | 14.18 | 88.45 | 12.01 |
| BRI-12449 | C(C1CN(c2ccccc12)S(C=Cc1ccc(cc1)[Cl])(=O)=O)C(O)=O | 377.0 | 2.8 | -3.4 | CysLT1R crystal structure | Z1233134481 | 67.50 | 16.88 | 67.85 | 10.01 |
| ***BRI-12450*** | ***CC(C)(C)C(Nc1ccc(cn1)c1ccc(C(N(C)CC(O)=O)=O)o1)=O*** | ***359.1*** | ***1.1*** | ***-2.3*** | ***CysLT2R crystal structure*** | ***Z2581545995*** | ***57.61*** | ***8.34*** | ***41.12*** | ***18.48*** |
| BRI-12451 | C1Cc2ccc(cc2C1)c1ccc(s1)S(NCC(O)=O)(=O)=O | 337.0 | 2.5 | -2.9 | CysLT2R crystal structure | Z2581538270 | 65.35 | 8.44 | 61.25 | 13.63 |
| BRI-12452 | C(C(Nc1ccc(cc1)c1nc2cccc(c2s1)[Cl])=O)c1ccc(cc1)S(CCO)(=O)=O | 486.0 | 4.0 | -4.3 | CysLT2R LiBERO-optimized model | Z3718279222 | 76.46 | 9.05 | 77.42 | 10.02 |
| *BRI-12453* | *C(C(N)=O)NC(c1ccc(CNC(C2=Cc3ccc(cc3NC2=O)F)=O)cc1)=O* | *396.1* | *0.9* | *-2.1* | *CysLT1R crystal structure* | *Z3718279199* | *88.32* | *8.31* | *49.49* | *14.31* |
| *BRI-12454* | *C1CN(CC(N1CC(N)=O)=O)C(c1ccccc1NC(c1ccc(cc1)[Cl])=O)=O* | *414.1* | *0.8* | *-2.5* | *CysLT2R crystal structure* | *Z3718279217* | *77.44* | *14.93* | *48.37* | *10.13* |
| BRI-12455 | C1CC(CN(C1)c1nc(cs1)c1ccccc1)n1cnc(C(N)=O)n1 | 354.1 | 2.6 | -2.7 | CysLT2R crystal structure | Z1754360775 | 84.21 | 8.39 | 71.11 | 15.00 |
| BRI-12456 | C(C(O)=O)N(CC(O)=O)S(Cc1cc(cc(c1)[Cl])[Cl])(=O)=O | 355.0 | -0.6 | -1.8 | CysLT1R LiBERO-optimized model | Z1738929033 | 96.31 | 14.52 | 65.24 | 19.66 |
| BRI-12457 | C1CN(C(N1)=O)c1ccc(cc1)C(Nc1cccc(c1)C(O)=O)=O | 325.1 | 2.4 | -3.1 | CysLT1R LiBERO-optimized model | Z364284232 | 83.79 | 16.97 | 67.44 | 10.61 |
| ***BRI-12458*** | ***CCc1c(c(N[C@@H]2C[C@@H](C2)CNC(c2ccn3cc(C)nc3c2)=O)ncn1)F*** | ***382.2*** | ***2.6*** | ***-2.9*** | ***CysLT1R LiBERO-optimized model*** | ***Z3718279213*** | ***51.34*** | ***13.05*** | ***40.92*** | ***18.79*** |
| BRI-12459 | C1[C@@H](C(N2CC3(CCN(C3)C(c3cc(C(F)(F)F)n[nH]3)=O)C2)=O)[C@@H]1c1cccnc1 | 419.2 | 1.7 | -1.4 | CysLT1R LiBERO-optimized model | Z3718279339 | 77.58 | 5.55 | 69.06 | 12.33 |
| BRI-12460 | C(C(c1cnn(c1)c1ccccc1)NS(Cc1cccc2cccnc12)(=O)=O)C(O)=O | 436.1 | 2.4 | -2.9 | CysLT2R LiBERO-optimized model | Z1738736877 | 83.87 | 17.49 | 73.50 | 9.45 |
| BRI-12461 | CN1C(Nc2ccc(cc12)C(N1CC(CNC(c2ccccc2OC)=O)C1)=O)=O | 394.2 | 1.6 | -2.4 | CysLT1R crystal structure | Z3718279208 | 87.64 | 12.34 | 58.44 | 16.77 |
| BRI-12462 | Cc1ccc2c(CC(N3CCN(CC3)C(c3ccc(C(N)=O)o3)=O)=O)c[nH]c2c1 | 394.2 | 1.4 | -2.0 | CysLT1R LiBERO-optimized model | Z3718279212 | 77.10 | 15.78 | 78.07 | 4.00 |
| BRI-12463 | Cc1c(CC(NC2(CCC2)CNC(c2cc3c(cco3)s2)=O)=O)non1 | 374.1 | 1.8 | -2.6 | CysLT2R crystal structure | Z3718279218 | 81.21 | 15.69 | 57.77 | 7.71 |
| BRI-12464 | CC(CNS(c1csc(c2ccc(cc2)F)n1)(=O)=O)C(O)=O | 344.0 | 2.3 | -2.8 | CysLT2R LiBERO-optimized model_final | Z1738076126 | 103.20 | 10.13 | 60.83 | 13.65 |
| BRI-12465 | C(CNC(c1c(C(Nc2ccc(cc2)[Cl])=O)nccn1)=O)C(O)=O | 348.1 | 0.4 | -2.2 | CysLT1R crystal structure | Z1455065943 | 84.70 | 13.98 | 59.92 | 11.02 |
| *BRI-12466* | *Cc1ccc2ncc(C(NC3CN(C3)C(c3cc(cs3)C(N)=O)=O)=O)n2c1* | *383.1* | *0.8* | *-2.0* | *CysLT1R crystal structure* | *Z3718279207* | *67.65* | *12.40* | *39.04* | *24.35* |
| BRI-12467 | C1Cc2ccc(cc2C1)C(NCC1(CN(C1)C(C1C=CC(NC=1)=O)=O)O)=O | 367.2 | 0.8 | -2.0 | CysLT1R crystal structure | Z3718279206 | 69.40 | 14.80 | 53.79 | 15.34 |
| BRI-12468 | COc1cccc(CC(N[C@H]2[C@@H]3CC[C@@H](C3)[C@H]2C(O)=O)=O)c1[Cl] | 337.1 | 3.0 | -3.3 | CysLT1R LiBERO-optimized model | Z2595765672 | 89.12 | 7.40 | 64.56 | 13.30 |
| BRI-12469 | CCOc1ccc(c(c1)C(N)=O)NC(c1cccn2c1nnn2)=O | 326.1 | 0.4 | -2.3 | CysLT1R LiBERO-optimized model | Z3718279215 | 80.65 | 11.17 | 52.82 | 19.19 |
| BRI-12470 | CCc1ccc(CN(C)S(c2c(cn[nH]2)C(O)=O)(=O)=O)cc1 | 323.1 | 2.5 | -2.8 | CysLT2R LiBERO-optimized model_final | Z928027252 | 80.95 | 9.80 | 58.60 | 14.11 |
| BRI-12471 | C(C(NCC(Nc1ccn(CC(O)=O)n1)=O)=O)c1cccc(c1)F | 334.1 | -0.6 | -2.0 | CysLT2R LiBERO-optimized model_final | Z1445119445 | 88.20 | 7.83 | 50.66 | 16.02 |
| BRI-12472 | CCC(Nc1ccc(cc1)S(NC(C)(C)CC(O)=O)(=O)=O)=O | 328.1 | 0.7 | -2.2 | CysLT1R LiBERO-optimized model | Z1744350205 | 95.84 | 15.47 | 66.40 | 9.25 |
| BRI-12473 | C(C(O)=O)NC(c1ccc(cc1)NC(COCc1ccccc1)=O)=O | 342.1 | 0.9 | -1.7 | CysLT1R crystal structure | Z85912191 | 86.50 | 17.34 | 66.62 | 16.04 |
| BRI-12474 | Cc1c(c2ccccc2)c(C(N)=O)c(NC(C(C)(CC(O)=O)C2CCCCC2)=O)s1.[Na] | 451.2 | 3.5 | -4.5 | CysLT1R LiBERO-optimized model | Z1024898266 | 117.50 | 5.89 | 106.30 | 9.22 |
| BRI-12475 | C(Cc1cccc(c1)NC(C1=CC=C(c2cccs2)NC1=O)=O)C(O)=O | 368.1 | 2.5 | -3.0 | CysLT2R LiBERO-optimized model | Z1946968027 | 68.34 | 7.08 | 74.60 | 10.69 |
| BRI-12476 | CC1(CCOCC1)C(Nc1ccc(cc1)C(NCCC(O)=O)=O)=O | 334.2 | 0.6 | -1.7 | CysLT2R LiBERO-optimized model_final | Z1444894063 | 90.10 | 8.48 | 66.41 | 13.27 |
| BRI-12477 | C[C@H](COC)N1C(=CSC1=Nc1ccc(cc1)C(N)=O)c1ccc2c(c1)OC(N2)=O | 424.1 | 2.5 | -3.0 | CysLT2R crystal structure | Z1569874527 | 74.90 | 8.13 | 60.63 | 18.67 |
| BRI-12478 | C(CNC(c1ccc(cc1)NC1c2ccccc2S(N=1)(=O)=O)=O)c1c[nH]c2cc(ccc12)[Cl] | 478.1 | 4.3 | -4.6 | CysLT2R LiBERO-optimized model | Z914145556 | 82.91 | 7.70 | 65.87 | 9.60 |
| Non-stimulated | | | | | | | -0.53 | 5.53 | 0.06 | 5.70 |
| LTD4 | | | | | | | 100.00 | 8.40 | 100.00 | 23.69 |
| LTD4 + Pranlukast | | | | | | | -13.97 | 7.09 | N/A | N/A |
| LTD4 + ONO-2080365 | | | | | | | N/A | N/A | -29.54 | 31.16 |

***Supplementary Table S6.*** ***Chemical similarity between experimental hits and known CysLTR ligands.***

| **BRI-ID** | **BRI structure** | **Structure of the most similar ligand from ChEMBL** | **Tanimoto distance** | **ChEMBL ID of ligand** | **Activity of the ligand from ChEMBL** |
| --- | --- | --- | --- | --- | --- |
| BRI-12359 |  |  | 0.57 | CHEMBL3809164 | IC50 (CysLT1) = 35nM |
| BRI-12410 |  |  | 0.62 | CHEMBL431348 | Kd (CysLT1) = 0.79nM |
| BRI-12411 |  |  | 0.54 | CHEMBL3342945 | IC50 (CysLT2) = 53nM |
| BRI-12417 |  |  | 0.54 | CHEMBL131611 | IC50 (CysLT1) = 1.5nM |
| BRI-12424 |  |  | 0.43 | CHEMBL131611 | IC50 (CysLT1) = 1.5nM |
